# Synthesis, Crystal Structure, Antitumor, and Antimicrobial Activity of Novel Copper(II) Complexes with a Coumarin Derivative Containing a Histamine Substituent

**DOI:** 10.3390/molecules31010162

**Published:** 2026-01-01

**Authors:** Ewelina Namiecińska, Pawel Hikisz, Patryk Czapnik, Magdalena Małecka, Magdalena Grazul, Peter Mayer, Ingo-Peter Lorenz, Elzbieta Budzisz

**Affiliations:** 1 Department of the Chemistry of Cosmetic Raw Materials, Medical University of Lodz, Muszynskiego 1, 90-151 Lodz, Poland; ewelina.namiecinska@umed.lodz.pl; 2 Department of Oncobiology and Epigenetics, Faculty of Biology and Environmental Protection, University of Lodz, Pomorska 141/143, 90-236 Lodz, Poland; pawel.hikisz@biol.uni.lodz.pl; 3 Department of Physical Chemistry, Faculty of Chemistry, University of Lodz, Pomorska 163/165, 90-236 Lodz, Poland; patryk.czapnik@edu.uni.lodz.pl (P.C.); magdalena.malecka@chemia.uni.lodz.pl (M.M.); 4 Doctoral School of Exact and Natural Sciences, University of Lodz, Jana Matejki 21/23, 90-237 Lodz, Poland; 5 Department of Pharmaceutical Microbiology and Microbiological Diagnostics, Faculty of Pharmacy, Medical University of Lodz, Muszynski 1 street, 90-151 Lodz, Poland; magdalena.grazul@umed.lodz.pl; 6 Department of Chemistry and Biochemistry, Ludwig Maximilians University, Butenandtstr. 5-13 (D), D-81377 Munich, Germany; pemay@cup.uni-muenchen.de (P.M.); ipb.lorenz@gmx.de (I.-P.L.)

**Keywords:** anticancer properties, copper(II) complex with coumarin ligands, crystal structures, flavonoid derivatives

## Abstract

Copper(II) complexes have great potential as antitumor and antimicrobial agents, and their coumarin derivatives bearing histamine substituents possess versatile structural and biological properties. The present article describes the synthesis of novel copper(II)–coumarin–histamine complexes and ligands and their characterization by IR, NMR, X-ray diffraction, and elemental analysis. Their antimicrobial activity (MIC, MBC/MFC) was tested against 11 reference strains. Cytotoxicity was evaluated using the MTT assay against 15 selected cancer cell lines and normal HMEC-1 cells. It presents three new ligands and three new complexes with copper(II) ions and selected histamine-containing coumarin derivatives. The new copper(II) complexes demonstrated markedly higher anticancer activity than their corresponding ligands across all evaluated cancer cell lines. The highest anticancer activity against the Hep3B liver cancer cell line was demonstrated by the copper(II) complex (**3b**), which also showed the strongest inhibition of *S. epidermidis* ATCC 12228 and *S. aureus* ATCC 6538. The copper(II) ions play a crucial role in the antitumor activity of these derivatives. Despite limited antimicrobial effects, the tested complexes, particularly **3a** and **3b**, demonstrate promising anticancer potential, especially against the Hep3B cancer cell line. Only **3b** demonstrated antimicrobial activity against *S. epidermidis* ATCC 12228 and *S. aureus* ATCC 6538.

## 1. Introduction

Infectious diseases and cancer continue to represent major global health challenges, and their treatment is driving the rapid development of innovative and effective therapeutic strategies. Although many antibiotics and chemotherapeutic agents are currently available, there is a constant need for compounds to accommodate the emergence of novel drug-resistant pathogens and infectious agents [1].

The human microbiome plays a crucial role in maintaining the integrity and homeostasis of the skin and mucosal barriers; it also influences the pathogenesis of numerous conditions, including chronic wounds, ulcers, and tumor-related infections. Patients undergoing or recovering from chemotherapy are particularly vulnerable to pathogenic microorganisms due to treatment-induced immunosuppression and the disruption of epithelial barriers, which substantially increases the risk of secondary infections [2]. Therefore, there is considerable interest in understanding the anticancer and potential antimicrobial activity of new compounds, particularly in patients with malignancies of the skin, breast, or head and neck, who are especially susceptible to wound infections [3,4,5]. Metal-based anticancer complexes, such as those containing Cu(II) or Ru(II) ions, have attracted considerable attention due to their multifaceted mechanisms of action. In addition to their cytotoxic effects on tumor cells, some of these complexes demonstrate auxiliary antimicrobial properties, which may reduce secondary infections during cancer treatment [5,6].

Chromone (benzo-γ-pyrone) derivatives and their analogues, including flavones (2-phenyl-4*H*-chromen-2-one), are well known for their wide-ranging anticancer, antioxidant, and anti-inflammatory effects [7,8]. Many of these compounds exhibit metal-chelating properties, enabling the formation of coordination complexes that often display enhanced biological activity [9]. Copper(II), a biologically relevant transition metal, plays an essential role in numerous enzymatic and redox processes and has been extensively investigated for its antitumor potential [10,11].

Chromane-2,4-dione derivatives, which are structurally related to coumarins, have emerged as promising scaffolds in medicinal chemistry due to their ability to undergo diverse structural modifications and interact with various biological targets [12,13]. When conjugated with heterocyclic moieties, such as amino-1H-imidazole or histamine derivatives, coumarin and chromane-2,4-dione analogues demonstrate notable pharmacological versatility, including anticancer, antibacterial, and anti-inflammatory properties [14,15,16]. Their coumarin-like structure can also influence coagulation pathways through vitamin K antagonism, adding another layer of biological relevance [17].

The present study describes the design and synthesis of novel coumarin (chromane-2,4-dione) derivatives containing histamine substituents, as well as their corresponding copper(II) complexes. The introduction of copper(II) ions was expected to enhance the biological activities of these ligands. Given the potential for dual functionality, the study evaluates both the cytotoxic and antimicrobial activities of the obtained compounds.

**Scheme 1:**
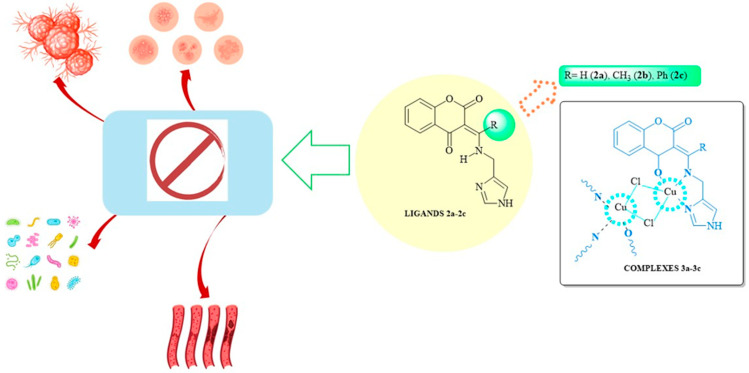
General structures of newly synthesized ligands **2a**–**2c** and their corresponding complexes **3a**–**3c**.

The aim of this study was to synthesize new complex compounds containing copper(II) ions with selected histamine-containing coumarin derivatives and to evaluate their anticancer and antimicrobial activity (Scheme 1).

## 2. Results

### 2.1. Synthesis of Compounds

**Scheme 2:**
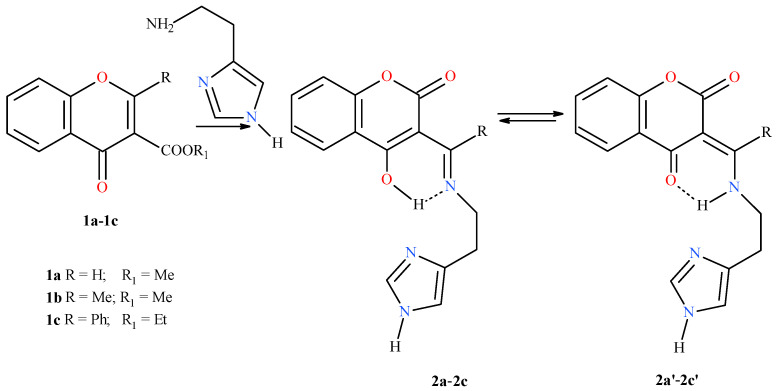
Synthesis of ligands **1a**/**2a**/**2a**’–**1c**/**2c**/**2c’**.

The starting materials, i.e., chromone derivatives (benzyl-γ-pyrone) **1a**–**1c**, were synthesized as described by Coppola et al. [18]. In the next step, histidine moieties were added by condensation of the appropriate chromone with histamine in a 1:1 molar ratio in a suitable solvent. A solution of histamine (0.1 mmol) in methanol (1 mL) was added dropwise to a stirred solution of the corresponding ester (**1a**–**1c**, 0.1 mmol) in methanol (10 mL). The mixture was stirred at room temperature for 24 h. The resulting ligands exist as tautomers **2a**–**2c** and **2a’**–**2c’** (Scheme 2).

Subsequently, the obtained derivatives **2a**–**2c** were subjected to complexation with copper(II) chloride in a 1:1 ratio, with ethanol or methanol (depending on the specific compound) as a solvent at room temperature.

**Scheme 3:**
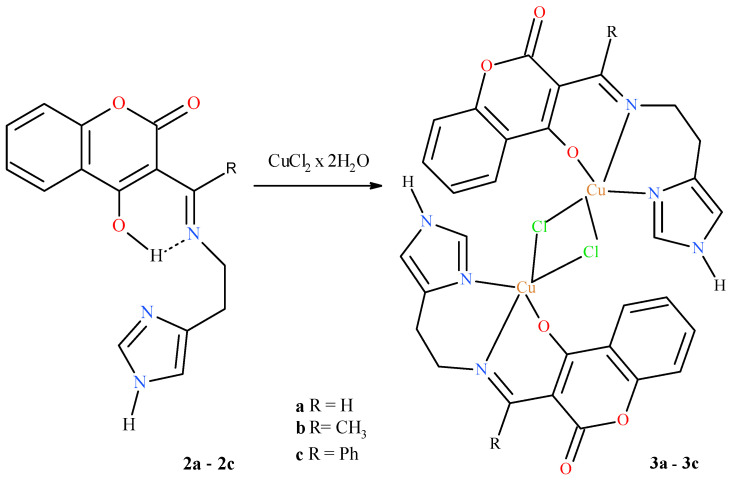
Synthesis of new copper(II) complexes **3a**–**3c** with coumarine derivatives **2a**–**2c**.

Copper(II) complexes **3a**–**3c** were prepared as follows. A solution of copper(II) chloride dihydrate (0.1 mmol) in methanol (1 mL) was added dropwise to a stirred solution of the corresponding ligand (**2a**–**2c**, 0.1 mmol) in methanol or ethanol (10 mL). The reaction mixture was stirred at room temperature for 24 h. The green to teal-blue precipitates of complexes **3a**–**3c** were filtered, washed with diethyl ether, and dried in air (Scheme 3).

The coumarin derivatives with histamine substituents, i.e., ligands **2a/2a’**–**2c/2c’**, and their copper(II) complexes **3a**–**3c** were characterized by spectroscopic methods (NMR for ligands only), IR spectroscopy, X-ray diffraction, ESI-MS, and elemental analysis. Their melting points were also determined.

### 2.2. Characterization of Ligand–Coumarin Derivatives **2a**–**2c** and Their Complexes **3a**–**3c**

#### The Selected Identification Methods

The ^1^H NMR spectral data were recorded in DMSO-d^6^ (for all ligands **2a**–**2c**), IR spectra in KBr (for all compounds **2a/3a**–**2c/3c**), and ES-MS spectra (for **3a**–**3c** compounds), as described in the Materials and methods section. The observed chemical shifts and intensities correspond well to those expected for the synthesized products. Aromatic protons were seen in the range from 7.29 to 7.93 ppm as multiplets, singlets, and doublets. The absorption bands characteristic of stretching vibrations of the histidine N–H groups were observed shifted to 3224 cm^−1^ and 3246 cm^−1^ (for ligands **2a**–**2c**) and to higher regions stretching from 3147 to 3108 cm^−1^ (for complexes **3a**–**3c**). Complexes of amino group protons in NMR spectra gave signals from 11.86 ppm to 13.67 ppm.

### 2.3. X-Ray Determination

#### The Structure of Ligand 2b

**Figure 1:**
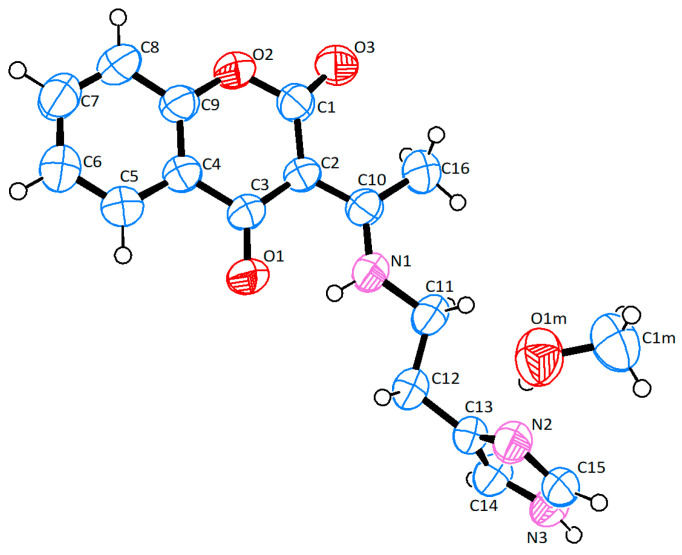
The molecular structure of compound **2b** (ellipsoid probability 50%).

The asymmetric unit of compound **2b** comprises one molecule of 3-(1-(((1*H*-imidazol-4-yl)methyl)amino)ethyl)chromane-2,4-dione and one molecule of methanol (Figure 1). The condensed chromone rings (atoms C1–C9 and O2) and the histidine ring (atoms C13, C14, N3, C15, N2) do not lie in the same plane: The angle between the planes through these atoms is 89.7°. Both rings are planar. The maximum deviation for the 10-membered ring of the best plane of the chromone moiety (through atoms C1–C9 and O2) is 0.0137 Å for O2, and the maximum deviation for the best plane of the 5-membered ring of histamine is 0.031 Å for N2. The bond lengths and angles are in Table 1. The crystal structure is stabilized by intermolecular and intramolecular hydrogen bonds (Table 2), e.g., the O1M–H1M...N2 hydrogen bond connects compound **2b** with a methanol molecule.

**Figure 2:**
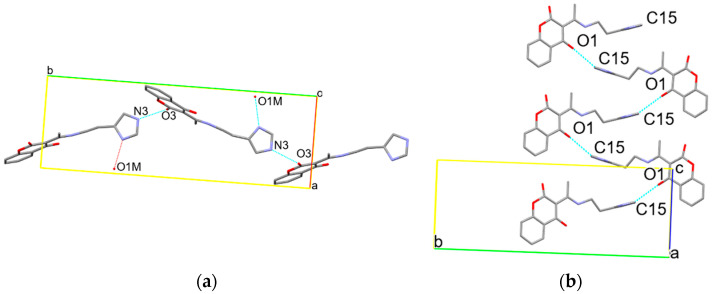
Two chains of the **2b** molecules along the b-axis (**a**) and along the c-axis (**b**).

**Figure 3:**
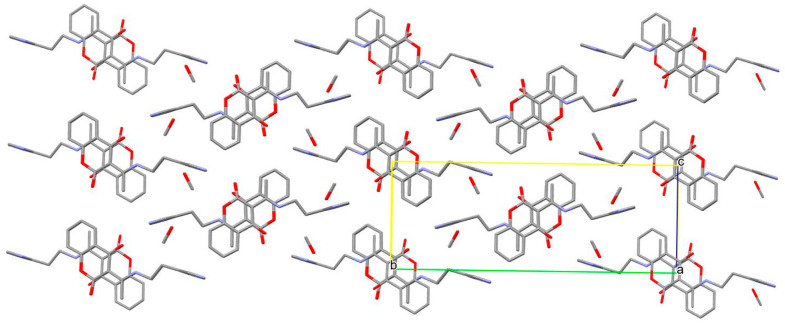
Arrangement of the **2b** molecules in the plane (010).

**Figure 4:**
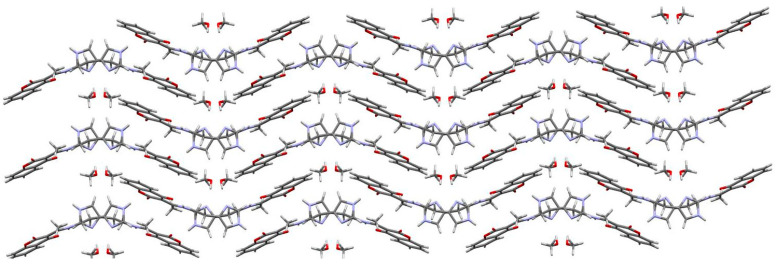
Arrangement of compound **2b** (viewed along 100).

The molecules of compound **2b** are connected by the hydrogen bond N3–H3...O3 (1−x, 1/2 + y, 5/2−z), forming a chain along the b axis. A chain motif can be described using the graph-set method as C(11) [19] (Figure 2a). The methanol molecules are attached to this chain via the O1M–H1M...N2 (x,y,−1 + z) bond. On the other hand, the C15–H15...O1(x,1/2−y,1/2 + z) bond creates another chain, C(11), propagating along the c-axis (Figure 2b). In the crystal lattice, the molecules are arranged to form a sheet in the (010) plane (Figure 3). The planes are stacked on top of each other in a “head-to-tail” arrangement, and the layers are repeated according to the ABAB scheme (Figure 4).

### 2.4. The Structure of the Dimeric Cu(II) Complex 3a

**Figure 5:**
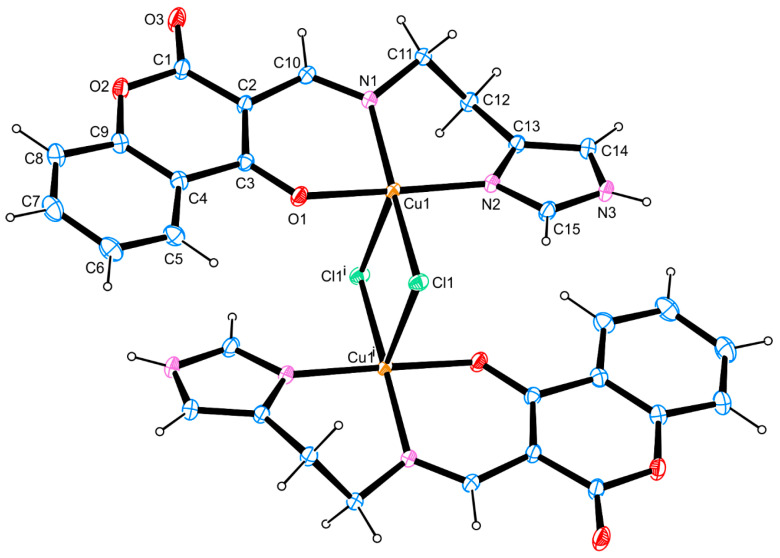
The molecular structure of the dimeric Cu(II) complex **3a**.

Figure 5 presents the molecular structure of the dimeric Cu(II) complex **3a**. The figures have been drawn at the 50% ellipsoid probability level. Symmetry code i = −x, 1−y, 1−z.

**Figure 6:**
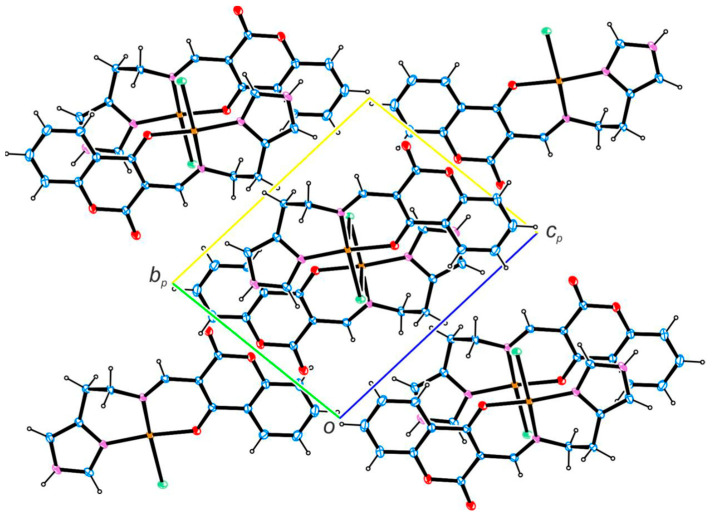
Crystal packing of the dimeric Cu(II) complex **3a**.

The copper(II) centers of the inversion-symmetric dinuclear complex 3a show a tetragonal pyramidal configuration of two nitrogen ligands (imido-N1 and -N2), one oxygen (O1), and two chlorido ligands, Cl1 and Cl1 (Figure 5). The angles within the plane around Cu1 lie between 85.02(3)° and 91.94(4)°. Their sum gives 358.63(4)°, and the four coordinated ligand atoms N1, N2, O1, and Cl1 demonstrate near planarity. The fifth ligand Cl1^i^, however, is situated in a slightly distorted pyramidal position, with its angles towards the basic ligands N1, N2, O1, and Cl1 between 91.85(3)° and 98.42(3)°. Due to the Jahn–Teller deformation of the central d^9^-ion, the Cu1–Cl1^i^ bond length (2.6668(4) Å) is significantly longer than that of Cu1–Cl1 (2.3555(3) Å). Both chloride ligands act as bridging ligands towards the second Cu1^i^ with the same but inverse configuration. The angle Cl1–Cu1–Cl1^i^ runs to 93.949(11)°, and that of Cu1–Cl1–Cu1^i^ to 86.051(11)°. The bond lengths Cu1–N1 (2.0200(11) Å) and Cu1–N2 (1.9783(11) Å) are only slightly different because of their sp^2^-bonding character and planar configuration. Both planar-surrounded atoms N1 and N2, however, show different bond lengths towards their different carbon neighbours (sp^2^ or sp^3^ hybridized), e.g., nearly equal for N2 (N2–C13 = 1.3860(17) Å and N2–C15 = 1.3332(16) Å), but different for N1 (N1–C10 = 1.2947(15) Å and N1–C11 = 1.4721(16) Å). The distance of Cu1 to the oxo ligand O1 is 1.9497(9) Å. The six-membered chelate ring of the bidentate N1, O1-containing ligand part around Cu1 is almost planar. Cu1 shows a maximum deviation of 0.102(1) Å from the least-square plane. The other six-membered chelate ring of the bidentate N1, N2-containing ligand part of Cu1 is folded by the influence of two sp^3^-hybridized atoms (C11 and C12). All bonds and angles are in Table 3. Figure 6 shows the crystal packing of the dimeric Cu(II) complex 3a.

### 2.5. Antimicrobial Activity

The compounds were tested against reference strains of *Staphylococcus aureus*, coagulase-negative staphylococci, and various representatives of other species known to be common sources of infection, including enterococci, *Enterobacter*, *Pseudomonas,* and *Proteus*. Vancomycin (VAN) was used as a reference agent against staphylococci and enterococci, and piperacillin–tazobactam (TZP) for Gram-negative rods. The antimicrobial activity of compounds **2a/3a**–**2c/3c** and antibiotics was expressed as minimum inhibitory concentrations (MIC) and minimum bactericidal concentrations (MBC) (see Supplementary Materials, Table S1).

Among all tested metal complexes, only complex **3b** indicated antimicrobial activity. While it only demonstrated activity against *S. epidermidis* ATCC12228 and *S. aureus* ATCC6538, its MIC and MBC values (78.6 µM) for staphylococcal strains were over 100 times higher than for vancomycin. All other combinations of agents, including **3b**, demonstrated MIC/MBC values higher than those for 500 µM against enterococci and all tested Gram-negative bacteria, indicating that they were inactive against the tested strains.

All tested ligands **2a**–**2c** were inactive against all tested strains, as their MIC/MBC values were over 500 µM.

### 2.6. Evaluation of Cell Viability by the MTT Spectrophotometric Method (Cytotoxicity Test)

The cytotoxic and antiproliferative properties of the six chromane-2,4-dione and imidazole derivatives were assessed using the MTT cytotoxicity assay. Anticancer studies were conducted on a broad panel of human cancer cells, utilizing as many as 15 cell lines differing in tumor type, molecular behavior, and genetics. Additionally, a single endothelial cell line was used as a model for normal cells. The cytotoxic effect was assessed after 24 h incubation with a wide range of test compound concentrations (0–500 µM). This experimental approach allows cells to undergo mitosis in the presence of the test compounds, thus providing insight into their inhibitory effects on cell proliferation. Cisplatin was used as a reference cytotoxic agent. Cell survival curves were generated for each ligand derivative; this was used to determine the IC50 value, i.e., the concentration required to inhibit cell proliferation by 50% compared to control cells. The IC50 results for each test compound against individual tumor cells are presented in Table 4.

The tested compounds generally exhibited low to negligible anticancer activity against the studied cancer cell lines. The calculated IC50 values for these derivatives were consistently 10 to 15 times higher than those of the reference (cisplatin) under identical experimental conditions. These results indicate that the compounds, in their current form, are not suitable as potent chemotherapeutic agents. However, some of the findings are promising and provide a solid foundation for the future synthesis and modification of these derivatives.

More specifically, compounds **2a**, **3a**, and **3b** showed higher activity against six intestinal cancer cell lines (HT-29, HCT116, COLO205, SW620, LoVo, and CaCo-2), causing a 50% decrease in cell viability within the approximate range of 50–90 µM, with a few exceptions. Interestingly, these same compounds were significantly less effective against female cancer cell lines (breast and endometrial cancer); this resistance was also evident in the cisplatin-resistant Hec-1-A cell line, where the tested derivatives failed to achieve better results.

The compounds demonstrated very little effect on the melanoma WM-115 and lung carcinoma A549 cell lines: The most active derivatives only caused a 50% reduction in cell viability at concentrations of approximately 145 µM. In contrast, the HL60 leukemia cell line was more sensitive, with all six tested derivatives showing reasonable IC50 values ranging from approximately 50–70 µM; however, even these values were still at least 10 times weaker than those of cisplatin on HL60 cells.

The most noteworthy results were obtained with compounds **3a**–**3c** against the liver cancer cell line Hep3b, the only cell line that showed greater sensitivity to these derivatives than to cisplatin. Compounds **3a** and **3b** demonstrated strong antiproliferative and cytotoxic effects, with IC50 values of approximately 10 µM (**3a**) and 8 µM (**3b**), making them roughly twice as potent as cisplatin. Compound **3c**, however, had a slightly higher IC50 value (approximately 10 µM higher) than cisplatin. These findings offer hope for the potential use of these derivatives in liver cancer treatment and suggest a clear path for further chemical modification to develop more active compounds.

## 3. Discussion

Coumarin and chromane-2,4-dione derivatives represent a promising class of biologically active compounds due to their structural flexibility and wide range of pharmacological aspects. Their biological activity, and particularly their anticancer effects, can be enhanced by coordination with transition metals such as copper(II), as documented previously [20,21,22].

Therefore, further studies on the anticancer properties of newly synthesized coumarin-based copper(II) complexes are needed to provide valuable and new information on their potential as multifunctional therapeutic agents. The present study evaluates the anticancer and antimicrobial activity of new complex compounds containing copper(II) ions with selected histamine-containing coumarin derivatives against selected bacterial strains and cancer cell lines. The structure of the derivatives was determined by spectroscopic methods, and their *N,N,O*-donor character, particularly that of the complex compound (**3a**), was verified by X-ray crystallographic analysis.

The structure of the synthesized derivatives offers important clues to their potential biological behavior, encouraging further studies into their anticancer and antimicrobial potential. The anticancer activity of coumarin derivatives coordinated with copper(II) ions has garnered considerable attention due to their diverse mechanisms of action and their capacity to achieve improved therapeutic profiles, characterized by increased efficacy and reduced side effects. However, to elucidate their anticancer and antimicrobial mechanisms, it is first necessary to understand the biochemistry of these compounds. These modifications can enhance aqueous solubility and bioavailability while simultaneously promoting selectivity for cancer cells over healthy cells [23,24].

Coordinating such ligands with transition metal ions, such as Cu(II), frequently enhances their biological potency, with the resulting complexes demonstrating significantly greater anticancer effects than the free organic ligands. Makowska et al. reported that a series of Cu(II) complexes of 2-iminocoumarins bearing triazine or benzoxazole/benzothiazole moieties exhibited potent cytotoxic activity against several human tumor cell lines, with IC_50_ values ranging from 0.04 to 15.66 µM, i.e., significantly better than those of the corresponding free ligands [25].

Similarly, synthesized coumarin-based acyl-hydrazone ligands complexed with Cu(II) (mononuclear or binuclear) exhibited strong cytotoxicity against cancer cell lines such as HepG-2 and MCF-7; in some cases, the value even surpassed that of cisplatin, while maintaining moderate toxicity toward normal cells [26].

Mechanistic investigations of Cu(II) complexes have found cytotoxicity to be mediated through various pathways, including DNA binding and intercalation, reactive oxygen species (ROS) induction, and mitochondrial dysfunction, as well as initiation of apoptosis by cell-cycle arrest, often at the G2/M checkpoint [27,28,29]. For example, a Cu(II) pyrazine-based complex was found to demonstrate significant cytotoxicity toward HeLa and other cancer cells (IC_50_ ≈ 17.5 µM), while sparing non-malignant cells; the mechanism was linked to oxidative stress and redox imbalance via upregulation of antioxidant defense genes [26]. Importantly, some of these Cu(II) complexes exhibit selectivity, showing high cytotoxicity toward cancer cells while sparing normal cells [30]. This supports the notion that coordination with copper can fine-tune the therapeutic window of coumarin-based compounds, making them more promising potential anticancer agents than their ligand precursors alone. It should also be noted that copper(II) complexes are known to exhibit dynamic behavior in aqueous and biological media, including possible ligand-exchange or partial dissociation processes. Therefore, under in vitro conditions, complexes **3a**–**3c** may undergo structural transformations, leading to the formation of copper-containing species different from the solid-state dimeric structures determined by X-ray crystallography. Consequently, the observed cytotoxic effects may result from a combination of intact complexes, partially dissociated species, and copper–ligand fragments formed in situ. Indeed, broader evidence suggests that Cu(II) coordination often significantly enhances cytotoxicity compared to free ligands. For instance, Cu(II) complexes of 2-iminocoumarin derivatives achieved very low micromolar IC_50_ values, sometimes outperforming cisplatin [25]. In other reported systems, Cu(II) complexes based on 1,10-phenanthroline or azole ligands induced apoptosis in cancer cells via ROS generation, mitochondrial depolarization, and DNA damage, while demonstrating fewer adverse effects on non-malignant cells [31].

Studies of derivatives incorporating various functional groups or substituents on the chromanone scaffold have identified the most effective structural motifs for enhancing bioactivity [32]. The compounds examined herein exhibited markedly higher anticancer activity than their corresponding ligands for all evaluated cancer cell lines, while maintaining negligible toxicity toward normal cells. Notably, the copper(II) complexes **3a** and **3b** showed significantly greater cytotoxicity against Hep3B cells (10.0 ± 2.1 µM and 8.1 ± 3.1 µM, respectively) compared with the reference drug cisplatin (22.8 ± 1.2 µM). Structurally, derivative **3a** differs from **3b** by a methyl substitution, which appears to have a significant impact on its activity toward Hep3B cells. Both demonstrated significantly better activity than complex **3c**, which contains a phenyl substituent (38.8 ± 2.9 µM).

The strong link between chemical structure and biological function underscores the need for continued synthetic optimization of these agents. Structure–activity relationship (SAR) studies show that specific substitutions within the chromane-2,4-dione core markedly influence anticancer activity. Modifying side chains can modulate apoptosis induction [33], while derivatives with electron-donating groups display notable cytotoxicity by influencing the cell cycle and apoptosis [33,34]. Likewise, introducing various substituents into the benzylidene moiety strongly alters biological efficacy [35]. Electron-withdrawing groups often enhance anticancer activity; the mechanism may be based on strengthening interactions with key proteins in cancer-related pathways and improving binding selectivity, which may also reduce off-target effects. These findings highlight the value of targeted synthesis for developing derivatives with superior therapeutic profiles. However, further in vitro and in vivo studies are needed to clarify their mechanisms of action and assess their clinical potential [36].

A stable human microbiome is essential for epithelial integrity. It is, however, often disrupted during chemotherapy, which predisposes cancer patients to opportunistic infections [37,38]. This creates a strong rationale for identifying compounds with both anticancer and antimicrobial properties. Although no studies have examined antimicrobial activity for copper(II) complexes with coumarin derivatives containing a histamine motif, some structurally related examples provide preliminary reference points.

Interestingly, our findings indicate that the highest anticancer activity against the Hep3B liver cancer cell line was demonstrated by copper(II) complex **3b**. It also showed the strongest inhibition of the growth of *S. epidermidis* ATCC 12228 and *S. aureus* ATCC 6538 strains, with MIC and MBC values of 78.6 µM (62 µg/mL). Notably, these values were approximately 100 times higher than those observed for vancomycin as a reference.

This observation aligns with previous reports on Cu(II) complexes of coumarin derivatives, which indicate that the antimicrobial profile varies considerably depending on the ligand structure and the microbial strain. For example, Cu(II) complexes of coumarin–thiadiazole ligands exhibited MIC values of 0.9–3.12 mg/mL against Gram-positive bacteria such as *S. aureus* and *S. epidermidis*, whereas the free ligands tended to be slightly more active [39]. Similarly, Cu(II) and Zn(II) complexes of 8-formyl-7-hydroxy-4-methylcoumarin ligands displayed MIC values of 25 μg/mL against *S. aureus* and 50 μg/mL against *B. subtilis* and *E. coli*, compared to 50–75 μg/mL for the ligand alone [40]. Furthermore, Cu(II) complexes of tridentate 2-(2-oxo-2*H*-chromene-substituted-yl)oxy acetic acids showed zones of inhibition of 14 mm against *C. albicans* and MIC = 0.110 μg/mL against *S. aureus*, whereas the free ligand was less potent [41]. In contrast, some Cu(II) complexes coordinated with Schiff base ligands derived from 8-acetyl-7-hydroxy-4-methylcoumarin and 3-amino-1,2,4-triazole exhibited little to no activity against certain Gram-positive strains, likely due to limited metal ion release [42].

Several important limitations of this study should be acknowledged. First, the assessment of anticancer activity was restricted to the MTT assay, which was used as a preliminary screening step in our research strategy. As the majority of the synthesized complexes exhibited only weak antiproliferative effects, i.e., with IC_50_ values typically more than tenfold higher than those of cisplatin, there was no biological justification at this stage to perform more advanced mechanistic analyses such as apoptosis detection, cell-cycle profiling, oxidative stress measurements, or clonogenic assays. Although such detailed experiments are essential for fully elucidating the mechanism of action, they have been reserved for future studies focusing on the few highly active candidates, such as complexes **3a** and **3b** against Hep3B cells. Consequently, the biological conclusions presented in this manuscript should be regarded as preliminary and exploratory.

Second, HMEC-1 endothelial cells were used as the model of normal, non-cancerous cells, which is consistent with the literature and with common practice in cytotoxicity screening of newly synthesized chemical compounds. Although HMEC-1 cells exhibit a relatively slow proliferation rate, it is important to emphasize that our experimental protocol included 24 h of exposure to the tested complexes, followed by 48 h of additional culture in fresh medium without copper complexes. This 72 h scheme ensured that HMEC-1 cells had sufficient time to undergo cell division and to manifest biologically relevant responses to the tested compounds despite their slower growth dynamics. Nevertheless, it is clear that more rapidly proliferating normal cell models, such as fibroblasts or mesenchymal stem cells (MSCs), could provide a more stringent and biologically informative reference point for future selectivity analyses.

Finally, the reference compound, cisplatin, displayed similar, or even lower, IC_50_ values in the normal HMEC-1 model compared to those observed for certain cancer cell lines. While this observation may initially appear biologically counterintuitive, it is not unprecedented and is consistent with literature reports indicating that cisplatin cytotoxicity is highly context-dependent and strongly influenced by the culture conditions, the intrinsic repair mechanisms of the cells, and the nature of the in vitro model. Nevertheless, such variability highlights the limitations of any single in vitro model and underscores the need for careful interpretation of reference-drug toxicity data.

Additionally, no speciation or stability studies were performed under physiological conditions; therefore, the exact chemical form of the copper(II) complexes present during the biological assays cannot be unambiguously defined at this stage.

These limitations collectively emphasize the necessity of expanding future research to include additional normal-cell models, as well as more in-depth mechanistic studies for the complexes demonstrating the most promising anticancer properties.

Overall, these findings may suggest that the antimicrobial efficacy of Cu(II) coumarin complexes is ligand dependent. This is also indicated by our present findings, with certain structural motifs, such as thiadiazole or chromene substitutions, favoring activity against Gram-positive opportunistic pathogens.

## 4. Materials and Methods

### 4.1. Materials

All obtained compounds were subjected to physicochemical analysis. All solvents (methanol, ethanol) used in this work were purchased from Merck (Sant Louis, MO, USA) and POCH (Gliwice, Poland) chemical companies and used without further purification. The infrared transmission spectra of crystalline products were recorded using a Nexus Thermo Nicolet FT-IR spectrophotometer (Thermo Nicolet, Waltham, MA, USA).

Elemental analyses were performed at the Faculty of Chemistry (University of Lodz) using a Vario Micro Cube Elemental analyzer (Langenselbold, Germany). ^1^H NMR spectra were recorded on a Bruker Avance III 600 MHz spectrometer (Bruker, Billerica, MA, USA). Both samples were dissolved in DMSO. Chemical shifts are given in ppm and coupling constants in Hz. Melting points were determined on a Büchii Melting Point B-540 apparatus (Büchi, Flawil, Switzerland) in capillary mode and were uncorrected. Mass spectrometry analyses were performed using a Synapt G2-Si q-TOF mass spectrometer (Waters) with an ESI source operating in both positive and negative ion modes. Accurate mass measurements were ensured using leucine enkephalin as a LockSpray™ reference. The optimized source parameters were as follows: capillary voltage 3.2 kV in positive ion mode and 2.7 kV in negative ion mode, cone voltage 40 V, source temperature 110 °C, desolvation gas (nitrogen) flow rate 600 L/h at a temperature of 350 °C, and nebulizer gas pressure of 6.5 bar. Samples were dissolved in DMSO and diluted with acetonitrile to 10 µg/mL prior to direct infusion at 25 µL/min. Anticancer activity was tested against several cancer cell lines: MCF-7, HCC38, HeLa, Ishikawa, HT-29, HCT116, COLO205, SW620, LoVo, CaCo-2, WM-115, HL60, Hep3B, A549, and normal cell line HMEC-1, carried out in the Department of Bioorganic Chemistry at the Medical University of Lodz (anticancer), while antimicrobial activity was carried out on selected bacterial strains were done in the Department of Pharmaceutical Microbiology and Microbiological Diagnostics at Medical University of Lodz.

### 4.2. Methods

#### 4.2.1. General Procedure for Compounds **2a**–**2c**

A solution of histamine (0.5 mmol) in methanol (1 mL) was added at room temperature to a solution of chromone derivatives **1a**−**1c** (0.5 mmol) in methanol (10 mL). The crude solid product, which precipitated after several minutes, was left at room temperature for 24 h; it was then filtered, dried, and recrystallized from methanol or other solvents. Compounds **2a**–**2c** were obtained as white or cream solids.

Synthesis of ligand 3-({[(1*H*-imidazol-4-yl)methyl]amino}methyl)-2*H*-1-benzopyran-2, 4 (3*H*)-dione (**2a**)

Yield: 42% Mp = 164.2–165.6 °C. ^1^HNMR (600MHz, DMSO-d^6^) δ (ppm): 2.88 (2H,t, 6Hz), 3.36 (s,3H), 3.86 (2H, d, 6Hz), 6.89 (s, 1H), 7.31 (s, 2H), 7.60 (s,2H), 8.33 (s,1H), 8.43 (dd, 1H), 11.70 (s, 1H), 11.89 (s, 1H). IR (KBr): υ (cm^−1^) 3227.88 (C-NH2), 3107.39, 2926.37, 2872.22 (C-H aliph), 1681.76 ν(C=O), 1612.86, 1569.55, 1487.33, 1467.66 (C=C), 1027.68 (C-O-C), 764.89 (C-H). Anal. calc for C_15_H_13_O_3_N_3_ (M = 283,31 g mol^−1^ (%): C 63.33; H 4.63; N 14.84. Found (%): C 63.59; H 4.47; N 14.83.

Synthesis of ligand 3-(1-{[(1*H*-imidazol-4-yl)methyl]amino}ethylidene)-2*H*-1-benzopyran-2, 4 (3*H*)-dione (**2b**)

Yield: 55% Mp = 162.3–162.6 °C. ^1^HNMR (600MHz, DMSO-d_6_) δ (ppm):2.89 (2H, t, 6 Hz), 3.32 (3H, s), 3.86 (3H, q, 6 Hz), 6.97 (s, 1H), 7.27 (m, 3H), 7.58 (m, 3H), 7.62 (m, 3H), 7.92 (1H, d, 12 Hz), 11.86 (s, 1H), 13.67 (s, 1H). IR (KBr): υ (cm^−1^) 3246.25 (C-NH2), 3193.43, 3062.37, 2953.58, 2832.99 (C-H aliph), 1642.31 ν(C=O), 1608.24, 1575.14, 1478.14, 1462.09, 1430.28 (C=C), 1063.46 (C-O-C), 762.51 (C-H). Anal. calc C_16_H_15_N_3_O_3_*H_2_O (M = 315,33 g mol^−1^) (%): C 60.94; H 5.43; N 13.33. Found (%): C 60.88; H 5.19; N 13.47.

Synthesis of 3-[{[(1*H*-imidazol-4-yl)methyl]amino}(phenyl)methyl]-2*H*-1-benzopyran-2, 4 (3*H*)-dione (**2c**)

Yield: 63,5% Mp = 134.8–135.4 °C.^1^HNMR (600MHz, DMSO-d^6^) δ (ppm): 2.75 (2H, t, 6 Hz), 3.35 (2H, t, 6 Hz), 7.57 (s, 1H), 7.62 (m, 7H), 7.63 (d, 1H), 7.64 (m,1H), 7.65 (s, 1H), 11.86 (s, 1H), 13.60 (s, 1H). IR (KBr): ʋ (cm^−1^) 3424.07 (C-NH2), 2974.60, 2877.43 (C-H aliph), 1711.16 (C=O), 1609.91, 1564.18, 1503.44, 1480.90, 1465.06 (C=C), 1028.82 (C-O-C), 759.24 (C-H). Anal. calc for C_21_H_18_N_3_O_4_ (M = 376.392 g mol^−1^) (%): C 67.01; H 4.82; N 11.17. Found (%): C 67,08; H 4.90; N 11.06.

#### 4.2.2. General Procedure for Compounds **3a**–**3c**

The appropriate chromone derivatives containing histamine substituents **2a**–**2c** (0.1 mmol) in methanol and ethanol (2 and 3 mL, respectively) was added at room temperature. A solution of copper(II) chloride dihydrate CuCl_2_2H_2_O (0.1 mmol) in methanol (1 mL) was added dropwise to a mixing solution of ligand. The reaction mixture was stirred at room temperature for 24 h, and the resulting precipitate was obtained. The solid was filtered, washed with diethyl ether, and dried in the air.

Synthesis of 3-({[(1*H*-imidazol-4-yl)methyl]amino}methyl)-2*H*-1-benzopyran-2, 4 (3*H*)-dione copper(II) complex (**3a**)

Yield: 68% Mp: dec < 252 °C. IR (KBr): ʋ (cm^−1^) 3108.57 (C-NH2), 3058.63, 2925.26 (C-H aliph), 1671.63, 1620.65, 1519.69, 1493.06, 1454.68 (C=C), 1034.60 (C-O-C), 759.04 (C-H). ESI- MS [m/z]: 727.00 [M − Cl]^+^. Anal. calc for C_30_H_26_N_6_O_6_Cl_2_Cu_2_ (M = 764.570 g mol^−1^) (%): C 47.13; H 3.42; N 10.99. Found (%): C 47.08; H 3.25; N 10.34.

Synthesis of 3-(1-{[(1*H*-imidazol-4-yl)methyl]amino}ethylidene)-2*H*-1-benzopyran-2, 4 (3*H*)-dione copper(II) complex (**3b**)

Yield: 62% Mp: dec < 188 °C. IR (KBr): ʋ (cm^−1^) 3221.03 (C-NH2), 3131.23, 2908.18, 2855.55 (C-H aliph), 1711.11, 1606.96, 1560.50, 1479.46, 1480.15 (C=C), 1078.09 (C-O-C), 754.68 (C-H). ESI- MS [m/z]: 755.00 [M − Cl]^+^. Anal. Calc. for C_32_H_28_N_6_O_6_Cl_2_Cu_2_ (M = 790.60 g*mol^−1^) anal. (%): C 48.75; H 3.33; N 10.66. Found (%): C 48.40; H 3.53; N 10.82.

Synthesis of 3-[{[(1*H*-imidazol-4-yl)methyl]amino}(phenyl)methyl]-2*H*-1-benzopyran-2, 4 (3*H*)-dione copper(II) complex (**3c**)

Yield: 55% Mp:dec < 164 °C. IR (KBr): ʋ (cm^−1^) 3147.19 (C-NH2), 3025.03, 2976.11, 2927.00 (C-H aliph), 1699.67, 1607.18, 1563.07, 1492.29, 1461.10 (C=C), 1027.94 (C-O-C), 761.94 (C-H). ESI- MS [m/z] for C_42_H_32_N_6_O_6_Cl_2_Cu_2_ (M = 914,74 g mol^−1^): 948.99 [(M + Cl]-; 455.01 [(M/2-H+]- (ESI-); 879.06 [M-Cl]+, 421.05 [M/2-Cl]+ (ESI+). Anal. calc for C_42_H_32_N_6_O_6_Cl_2_Cu_2_ *3.8H_2_0 (M = 983.206 g mol^−1^) (%): C 51.31; H 4.06; N 8.55. Found (%): C 51.33; H 4.01; N 8.49.

#### 4.2.3. Refinement of X-Ray Data

The X-ray intensity data of **2b** and **3a** were measured on a XtaLAB Synergy S, Duaflex, HyPix Rigaku, and Bruker D8 Venture TXS system, respectively. The CuKα (micro-focus) wavelength was used for compound **2b**, and the MoKα (rotating anode) for **3a**. The frames were integrated with CrysAlisPro [43] (**2b**) and the Bruker SAINT software (23.1) package (**3a**) [44]. Data for **3a** were corrected for absorption effects using the Multi-Scan method (SADABS) [45]. The structures were solved and refined using the SHELXTL Software Package (2014) [46]. All C-bound hydrogen atoms were calculated in ideal geometry riding on their parent atoms, while the N-bound hydrogen atoms were refined freely.

The crystallographic data were deposited with the Cambridge Crystallographic Data Centre (CCDC), 12 Union Road, Cambridge CB21EZ, UK. Copies of the data can be obtained free of charge on quoting the depository numbers CCDC-2499734 (**2a**) and CCDC-2497425 (**3a**) (https://www.ccdc.cam.ac.uk/structures/, accessed on 28 October 2025). Crystal data and structure refinement for **2b** and **3a** are shown in Table 5.

#### 4.2.4. Preparation of Tested Compounds and Antibiotics

The antibacterial activity of tested metal complexes and their ligands was evaluated using the broth microdilution method, based on EUCAST recommendations [47] and expressed as MIC (minimum inhibitory concentration) and MBC (minimal bactericidal concentration) values. Vancomycin (Van) and piperacillin–tazobactam (TZP) were used as references. All antibiotics were purchased from Oxoid (Thermo Scientific). All stock solutions of tested compounds **2a**–**2c** and **3a**–**3c** were freshly prepared in 100% DMSO (POCh, Gliwice, Poland), while antibiotics were added directly to the Mueller–Hinton broth. The final concentration of DMSO in samples never exceeded 0.01%. The tested compounds were subjected to a two-fold dilution series prepared in Mueller–Hinton broth: 7.8–1000 µg/mL. All experiments were performed in triplicate.

##### Tested Bacterial Strains and Culture Conditions

The antibacterial activity of all compounds was tested against 11 reference strains: *S. epidermidis* ATCC 12228*, S. aureus* ATCC 6538, *P. vulgaris* CCM 1799, *P. aeruginosa* ATCC 15442, *four E. faecalis strains:* ATCC 29212, ATCC 129, PCM 896, PCM 1859, and *three E. coli strains:* ATCC 8739, ATCC 10539, and ATCC 25922. All tested bacterial strains were grown for 24 h in 35–37 °C on tryptic-soy agar (TSA; BTL Sp. z o.o., Łódź, Poland). Microbial suspensions at a density of about 5 × 10^5^ CFU/mL were prepared in Mueller–Hinton broth (MHB; BTL Sp. z o.o., Lodz, Poland).

### 4.3. Anticancer Activity

#### 4.3.1. Biological Material, Culture, and Passage of Cells

The cytotoxicity of the tested chromane-2,4-dione and imidazole compounds was determined in vitro against selected cancer cell types. These included six human adherent colon cancer lines: HCT 116 (ATCC^®^ CCL-247™, colorectal cancer, Dukes’ type A), SW620 (ATCC^®^ CCL-227™, colorectal adenocancer, Dukes’ type C), LoVo (ATCC^®^ CCL-229™, colorectal adenocancer, Dukes’ type C, grade IV), Caco-2 (ATCC^®^ HTB-37™, colorectal adenocancer, Dukes’ type B), HT-29 (ATCC^®^ HTB-38™, colorectal adenocancer, Dukes’ type C), Colo205 (ATCC^®^ CCL-222™, colon adenocarcinoma). Two breast epithelial adenocarcinoma cell lines, i.e., of significance in women, were included: one positive for estrogen and progesterone receptors, MCF-7 (ATCC^®^ HTB-22™), and one negative for estrogen and progesterone receptors, HCC38 (ATCC^®^ CRL-2314™). In addition, two endometrial cancer lines were used: Ishikawa (99040201; Sigma-Aldrich, St. Louis, MO, USA) and Hec-1-A (ATCC^®^ HTB-112™), and the cervical adenocarcinoma HeLa cell line (ATCC^®^ CCL-2™). Additionally, the studies used human lung cancer A549 line (ATCC^®^ CCL-185™, lung carcinoma), the promyelocytic leukemia HL60 line (ATCC^®^ CCL-240™, promyelocytic leukemia), hepatocellular carcinoma Hep3B line (ATCC^®^ HB-8064), and the malignant melanoma WM-115 cell line (91061232, human skin, epithelial).

All but one cell line were purchased from American Culture Collection (ATCC), Manassas, VA, USA; the WM-115 line was purchased from Sigma-Aldrich (Sigma-Aldrich; St. Louis, MO, USA). The selection of cell lines was dictated by genetic differences and the diversity of molecular subtypes of individual cell lines. The HMEC-1 (ATCC^®^ CRL-3243™) line was used as a model of normal cells.

HCT 116, SW620, LoVo, HT-29, Colo205, MCF-7, Ishikawa, Hec-1-A, WM-115, A549, and HeLa cancer cell lines were cultured in Dulbecco’s minimal essential medium (DMEM, Lonza, Visp, Switzerland) supplemented with 10% fetal bovine serum and antibiotics (10 U/mL penicillin and 0.5 mg/mL streptomycin). The Caco-2 cell line was cultured in Eagle’s minimum essential medium (EMEM, Sigma-Aldrich; St. Louis, MO, USA) supplemented with 20% fetal bovine serum and antibiotics (10 U/mL penicillin and 0.5 mg/mL streptomycin). HL60 and HCC38 tumor cells were cultured in RPMI (Thermo Fisher Scientific, Waltham, MA, USA) culture medium supplemented with 10% fetal bovine serum and antibiotics (10 U/mL penicillin and 0.5 mg/mL streptomycin). HMEC-1 cells were cultured in MCDB 131 (Corning Life Sciences, Corning, NY, USA) culture medium supplemented with 10% fetal bovine serum (Gibco, Grand Island, New York, NY, USA) and antibiotics (10 U/mL penicillin and 0.5 mg/mL streptomycin) (Gibco, Grand Island, New York, NY, USA). The culture medium was enhanced with *L*-glutamine (10 M), hydrocortisone (1 μg/mL), and epidermal growth factor (EGF; 10 ng/mL) (Millipore, Burlington, MA, USA).

Cell cultures were maintained under sterile conditions in sterile, disposable culture vessels. The cells were cultured in a standard CO_2_ incubator (37 °C, 95% air, 5% CO_2_, and 100% relative humidity). All cell lines were kept in the logarithmic growth phase by performing regular passages (2–3 times per week) to new culture vessels once the culture had reached approximately 80% confluence.

The procedure for passaging was as follows: The culture medium was first removed, and the cell monolayer was washed with 0.9% NaCl solution. Then, 0.25% trypsin solution with EDTA (Gibco, Grand Island, New York, NY, USA) was added in an amount appropriate for the surface of the specific culture vessel (300–500 μL). The cells were incubated with trypsin for 3–5 min in a CO_2_ incubator, and the trypsinization process was monitored with a microscope. After the cells had detached, culture medium was added in a volume appropriate for the size of the new vessel. The suspension was thoroughly mixed and transferred to new, sterile culture vessels. Finally, cells from each line were seeded onto culture plates or dishes at a density appropriate for the specific experiment.

#### 4.3.2. In Vitro Assessment of Cell Viability Using the MTT Assay

Cell viability and proliferation were assessed using the MTT (Methylthiazolyl diphenyltetrazolium bromide) assay, a metabolic microplate spectrophotometric method that employs the tetrazolium salt 3-(4,5-Dimethyl-2-thiazolyl)-2,5-diphenyl-2*H*-tetrazolium bromide (Sigma-Aldrich; St. Louis, MO, USA). This assay was utilized to evaluate the anti-proliferative activity of various compounds against different cancer cell lines.

For the assay, cells were seeded into 96-well microplates at a density of 6–8 × 10^3^ cells/mL. Following a 24 h incubation period to allow for cell attachment, test compounds were administered at a concentration range of 10–500 μmol/L. The molar concentrations used in the study were calculated based on the molar masses of the synthesized dimeric complexes in their solid form. It should be emphasized that, due to the dimeric nature of complexes **3a**–**3c**, one molar equivalent of a copper(II) complex corresponds to two molar equivalents of the coordinated ligand. This stoichiometric relationship was taken into account when interpreting the cytotoxicity data and comparing the biological activity of the complexes with that of the free ligands. The plates were then incubated for an additional 24 h. After this exposure period, the treatment media were removed, and the cell monolayers were washed twice with sterile phosphate-buffered saline (PBS) before being replenished with fresh culture medium. The cells were grown for another 48 h in fresh medium.

The MTT assay itself was performed 48 h after the initial 24 h treatment, using a 24 h incubation with the tested derivatives: This duration provides sufficient exposure to the compounds to trigger early cytotoxic mechanisms while minimizing potential adverse effects associated with prolonged treatment. After the 24 h treatment, the medium containing the compounds was removed, and the cells were allowed to recover in fresh medium for an additional 48 h. This recovery period enables the manifestation of both immediate and delayed cytotoxic effects, including the loss of proliferative capacity. This treatment scheme allows for a more comprehensive assessment of the compounds’ effects on cell viability in the MTT assay. A 50 μL aliquot of MTT solution (final concentration of 0.05 mg/mL) was added to each well, and the microplates were incubated for 3–4 h. During this incubation period, the yellow MTT was converted into purple formazan crystals by metabolically active cells; 100 μL of dimethyl sulfoxide (DMSO; Sigma-Aldrich, St. Louis, MO, USA) was then added to each well to solubilize the crystals.

The absorbance of the resulting formazan solution was measured using a Biotek Power Wave HT microplate reader (Biotek Instruments, Winooski, VT, USA) at a primary wavelength of λ = 580 nm, with a reference wavelength of λ = 720 nm to correct for background noise. The percentage of viable cells was calculated by normalizing the absorbance of the treated wells to that of the untreated control wells, which were considered to have 100% viability. The half-maximal inhibitory concentration (IC_50_), defined as the concentration of a compound required to reduce cell viability by 50%, was subsequently calculated. Cisplatin (Sigma-Aldrich, St. Louis, MO, USA) was included as a positive control (reference compound), and its inhibitory activity was evaluated under identical experimental conditions.

## 5. Conclusions

Coumarin derivatives with a histamine substituent as ligands merit attention with regard to both their structure and biological activity. The cytotoxic activity tested for copper(II) complexes in comparison to their corresponding ligands indicates that, in the vast majority of the cell lines tested, **3a**–**3c** derivatives proved to be biologically much more attractive than their corresponding ligands. Particularly high antiproliferative activity was observed for the Hep3b line, with the in vitro test data indicating that the copper(II) ions play a crucial role in this antitumor activity. In fact, some analyzed derivatives demonstrated lower IC_50_ values than the reference drug cisplatin, indicating their promising anticancer potential for this particular cancer line.

Despite their pronounced cytotoxic activity, the tested ligands and metal complexes generally demonstrated poor antimicrobial properties; however, complex **3b** showed weak activity limited to *S. aureus* ATCC6538 and *S. epidermidis* ATCC12228 strains. Hence, the tested compounds can be primarily selective toward cancer cells rather than microbial targets.

Nevertheless, deeper mechanistic in vitro and in vivo studies are required to fully elucidate the pharmacological profiles and safety of the derivatives for potential clinical applications.

## Figures and Tables

**Table 1:** Selected bond lengths [A] and angles [°] of compound **2b**.

Bonds		Angles	
O1–C3	1.2533(1)	C1–O2–C9	122.52(1)
O2–C1	1.3836(1)	C10–N1–C11	128.28(1)
O2–C9	1.3701(1)	C13–N2–C15	104.91(1)
O3–C1	1.2090(1)	C14–N3–C15	107.48(1)
N1–C10	1.3018(1)	O2–C1–O3	112.95(1)
N1–C11	1.4678(1)	O2–C1–C2	118.60(1)
N2–C13	1.3722(1)	O3–C1–C2	128.45(1)
N2–C15	1.3216(1)	O1–C3–C2	123.69(1)
N3–C14	1.3688(1)	O1–C3–C4	118.59(1)
N3–C15	1.3123(1)	O2–C9–C4	121.50(1)
C1–C2	1.4392(1)	O2–C9–C8	117.05(1)
C2–C3	1.4272(1)	N1–C10–C2	118.48(1)
C2–C10	1.4290(1)	N1–C10–C16	118.05(1)
C3–C4	1.4689(1)	N1–C11–C12	109.53(1)
C4–C5	1.3851(1)	N2–C13–C12	120.64(1)
C4–C9	1.3729(1)	N2–C13–C14	109.51(1)
C5–C6	1.3801(1)	N3–C14–C13	106.06(1)
C6–C7	1.3748(1)	N2–C15–N3	112.04(1)
C7–C8	1.3652(1)		
C8–C9	1.3843(1)		
C10–C16	1.4896(1)		
C11–C12	1.5097(1)		
C12–C13	1.4834(1)		
C13–C14	1.3469(1)		

**Table 2:** Geometry of hydrogen bonds stabilizing the **2b** structure.

D–H···A	D–H	H···A	D···A	D–H···A	Symmetry
N1-H1...O1	0.86	1.82	2.5510(1)	142	Intra
N3-H3...O3	0.86	2.05	2.8495(1)	154	1 − x,1/2 + y,5/2 − z
O1M-H1M...N2	0.82	1.94	2.7587(1)	173	x,y, −1 + z
C8-H8…O1M	0.93	2.59	3.3493(2)	140	−x, −y,1 − z
C14-H14...O1M	0.93	2.38	3.2176(2)	149	1 + x,y,1 + z
C15-H15...O1	0.93	2.39	3.2506(2)	154	x,1/2 − y,1/2 + z

**Table 3:** Selected bond lengths [A] and angles [°] of the **3a** complex.

Bonds		Angles	
Cu1–Cl1	2.3555(3)	N1–Cu1–O1	90.22(4)
Cu1–N1	2.0200(11)	N1–Cu1–N2	91.94(4)
Cu1–N2	1.9783(11)	N1–Cu1–Cl1	166.89(3)
Cu1–O1	1.9497(9)	O1–Cu1–N2	173.06(4)
Cu1–Cl1	2.6668(4)	N1–Cu1–Cl1	98.42(3)
N1–C10	1.2947(15)	N2–Cu1–Cl1	94.36(3)
N1–C11	1.4721(16)	O1–Cu1–Cl1	91.85(3)
N2–C13	1.3860(17)	Cl1–Cu1–Cl1	93.949(11)
N2–C15	1.3332(16)	Cu1–Cl1–Cu1	86.051(11)
		Cu1–N1–C10	124.08(9)
		N1–C10–C2	126.93(11)
		C10–C2–C3	122.63(11)
		Cu1–O1–C3	129.26(9)
		Cu1–N1–C11	121.36(8)
		Cu1–N2–C13	125.31(8)
		N1–C11–C12	109.99(10)
		C11–C12–C13	109.71(10)
		C12–C13–N2	120.94(11)

**Table 4:** In vitro cytotoxicity of newly synthesized ligands **2a**–**2c** and their corresponding complexes **3a**–**3c**, with cisplatin as a reference compound, against various human cancer cells ^1^ and the immortalized human microvascular endothelial cell line HMEC-1 as a model of normal cells. The cells were incubated with the test compounds for 24 h at concentrations of 0–500 µM. After this period, the medium containing the test compounds was removed, and the cells were grown for another 48 h in fresh medium. After this period, MTT cytotoxicity was measured. For copper(II) complexes **3a**–**3c**, the reported IC_50_ values refer to the molar concentration of the dimeric complex; thus, each value corresponds to two ligand units per complex molecule. Results are expressed as mean ± SEM of three repeated experiments. IC_50_ values (μM) indicate the concentration of a tested compound needed to reduce the fraction of surviving cells to 50% of that observed for the control sample (untreated cells).

Cell Line	Cytotoxic Effect of Chromane-2,4-Dione and Imidazole Compounds, and the Reference Compound Cisplatin, IC50 (μM)						
	**2a**	**2b**	**2c**	**3a**	**3b**	**3c**	**Cisplatin**
MCF-7	80.4 ± 1.4	129.5 ± 1.9	90.4 ± 3.2	52.1 ± 3.4	84.2 ± 3.9	67.8 ± 6.4	7.5 ± 1.1
HCC38	82.3 ± 2.4	>148.7 ± 4.4	79.6 ± 2.2	56.5 ± 1.8	40.1 ± 4.4	43.2 ± 2.6	3.1 ± 0.8
HeLa	155.5 ± 3.8	>500	99.4 ± 3.4	95.2 ± 6.1	175.1 ± 7.4	98.8 ± 7.1	20.5 ± 2.7
Hec-1-A	73.3 ± 1.9	84.7 ± 2.7	90.5 ± 4.4	83.9 ± 4.9	83.2 ± 3.4	81.1 ± 7.1	63.4 ± 2.1
Ishikawa	74.2 ± 3.9	121.6 ± 6.3	110.2 ± 3.2	72.7 ± 4.4	86.6 ± 6.2	69.9 ± 1.7	12.5 ± 2.3
HT-29	52.1 ± 2.4	>500	232.2 ± 8.1	51.9 ± 2.1	71.3 ± 3.1	79.5 ± 7.1	125.6 ± 1.3
HCT116	61.3 ± 4.1	77.1 ± 4.4	70.4 ± 1.1	80.7± 4.1	75.7 ± 4.4	92.5 ± 5.1	8.4 ± 1.1
COLO205	62.1 ± 6.6	>500	198.7 ± 6.4	93.4 ± 3.9	54.2 ± 5.1	79.3 ± 2.14	21.5 ± 1.7
SW620	119.2 ± 2.2	332.1 ± 7.1	221.4 ± 2.7	162.9 ± 2.5	71.4 ± 5.2	145 ± 2.8	7.4 ± 1
LoVo	63.6 ± 1.2	169.1 ± 5.8	125.6± 7.7	35.5± 5.6	42.5 ± 6.4	53.6 ± 4.2	8.6 ± 0.9
CaCo-2	91.4 ± 4	174 ± 2.9	95.2 ± 3.1	65.8 ± 5.1	60.7 ± 4.7	65.2 ± 4.8	5.4 ± 1.4
WM-115	143.3 ± 1.6	>500	324.5 ± 4.2	110.4 ± 3.1	213.8 ± 1.9	201.6 ± 2.8	15.4 ± 1
HL60	51.6 ± 1.9	63.4 ± 1.2	68.7 ± 2.8	42.1 ± 2.9	66.3 ± 2.2	68.5 ± 6.7	4.2 ± 0.8
Hep3b	69.7± 5.8	40.3 ± 7.1	52.6 ± 1.9	10 ± 2.1	8.1 ± 3.1	38.8 ± 2.9	22.8 ± 1.2
A549	261.7 ± 6.9	>500	243.4 ± 5.2	175.2 ± 4.9	145.2 ± 4.6	279.8 ± 6.4	46.6 ± 3.4
HMEC-1	167.2 ± 1.7	>500	211.2 ± 4.9	168.7 ± 7.1	112.7 ± 5.1	156.7 ± 4	11.7 ± 0.9

^1^ Breast cancer cell lines: MCF-7, HCC38; cervical cancer cell line: HeLa; endometrial cancer cell lines: Hec-1-A, Ishikawa; colon cancer cell lines: HT-29, HCT116, COLO205, SW620, LoVo, and CaCo-2; melanoma cell line: WM-115; leukemia cell line: HL60; hepatocellular carcinoma cell line: Hep3b; lung cancer cell line: A549.

**Table 5:** Crystal data and structure refinement for **2b** and **3a.**

	**2b**	**3a**
net formula	C_17_H_19_N_3_O_4_	C_30_H_24_Cl_2_Cu_2_N_6_O_6_
*M*_r_/g mol^−1^	329.35	762.53
crystal size/mm	0.231 × 0.175 × 0.036	0.100 0.040 × 0.020
*T*/K	294	102.(2)
radiation	CuKα	MoKα
diffractometer	‘XtaLAB Synergy, Dualflex, HyPix’	‘Bruker D8 Venture TXS’
crystal system	monoclinic	triclinic
space group	‘P2_1_/c’	‘P -1’
*a*/Å	7.9524(4)	7.1965(2)
*b*/Å	23.2722(9)	9.0330(3)
*c*/Å	8.7869(4)	11.6841(4)
α/°	90	95.1950(10)
β/°	93.344(5)	106.2340(10)
γ/°	90	98.5700(10)
*V*/Å^3^	1623.43(13)	714.08(4)
*Z*	4	1
calc. density/g cm^−3^	1.348	1.773
μ/mm^−1^	0.807	1.735
absorption correction	Multi-Scan	Multi-Scan
transmission factor range	0.551–1.000	0.88–0.97
refls. measured	24714	17067
*R*_int_	0.01	0.0365
mean σ(*I*)/*I*	0.0590	0.0471
θ range	3.799–77.269	2.998–36.318
observed refls.	1625	5753
*x, y* (weighting scheme)	0.1227, 0	0.0192, 0.4084
hydrogen refinement	rigid body model	mixed
refls in refinement	3274	6852
parameters	220	212
restraints	0	0
*R*(*F*_obs_)	0.0664	0.0315
*R*_w_(*F*^2^)	0.1351	0.0777
*S*	0.985	1.068
shift/error_max_	0	0.001
max electron density/e Å^−3^	0.202	0.702
min electron density/e Å^−3^	−0.247	−0.627

## Data Availability

The original contributions presented in this study are included in the article/supplementary material. Further inquiries can be directed to the corresponding author(s).

## Supplementary Materials

The following supporting information can be downloaded at: https://www.mdpi.com/article/10.3390/molecules31010162/s1, Table S1: The antimicrobial activity (expressed as MIC and MBC) of metal complexes 3a–3c and chosen antibiotics; Figures S1–S3: Spectrum FTIR (KBr cm-1) for compound 2a–2c; Figures S4–S6: Spectrum 1H NMR (600 MHz, DMSO-d6) for compounds 2a–2c; Figures S7–S9: Spectrum FTIR (KBr cm-1) for compound 3a–3c; Figure S10a: ESI mass spectra of compound 3a in positive ion mode; Figure S11a: ESI mass spectra of compound 3b in negative ion mode; Figure S11b: ESI mass spectra of compound 3b in positive ion mode; Figure S11c: ESI mass spectra zoom of views of target ions for compound 3b in positive ion mode; Figure S12a: ESI mass spectra of compound 3c in negative ion mode with the experimentally observed peak at m/z 948; Figure S12b: ESI mass spectra of compound 3c in negative ion mode with the experimentally observed peak at m/z 455; Figure S12c: ESI mass spectra of compound 3c in negative ion mode with the experimentally observed peak at m/z 421; Figures S13 and S14: The simulated isotope patterns of the corresponding compounds 3a–3b; Figure S15a: The simulated isotope patterns of the corresponding compound 3c after loss of crystallization water; Figure S15b: The simulated isotope patterns of the corresponding ion of compound 3c, after loss of crystallization water for the monomer under ESI.

## References

[1] Fromantin I., Watson S., Baffie A., Rivat A., Falcou M.C., Kriegel I., Ingenior Y. (2014). A Prospective, Descriptive Cohort Study of Malignant Wound Characteristics and Wound Care Strategies in Patients with Breast Cancer. Ostomy Wound Manag..

[2] Delgado A., Guddati A.K. (2021). Infections in hospitalized cancer patients. World J. Oncol..

[3] Payne W.G., Naidu D.K., Wheeler C.K., Barkoe D., Mentis M., Salas R.E., Smith D.J., Robson M.C. (2008). Wound healing in patients with cancer. Eplasty.

[4] Nesher L., Rolston K.V.I. (2014). The current spectrum of infections in cancer patients with chemotherapy related neutropenia. Infection.

[5] Hangan A.C., Lucaciu R.L., Turza A., Dican L., Sevastre B., Páll E., Oprean L.S., Borodi G. (2023). New Copper Complexes with Antibacterial and Cytotoxic Activity. Int. J. Mol. Sci..

[6] Balewski Ł., Plech T., Korona-Głowniak I., Hering A., Szczesio M., Olczak A., Bednarski P.J., Kokoszka J., Kornicka A. (2024). Copper(II) Complexes with 1-(Isoquinolin-3-yl)heteroalkyl-2-ones: Synthesis, Structure and Evaluation of Anticancer, Antimicrobial and Antioxidant Potential. Int. J. Mol. Sci..

[7] Keri R.S., Budagumpi S., Pai R.K., Balakrishna R.G. (2014). Chromones as a privileged scaffold in drug discovery: A review. Eur. J. Med. Chem..

[8] Kamboj S., Singh R. (2021). Chromanone—A prerogative therapeutic scaffold: An overview. Arab. J. Sci. Eng..

[9] Rodríguez-Arce E., Saldías M. (2021). Antioxidant properties of flavonoid metal complexes and their potential inclusion in the development of novel strategies for the treatment against neurodegenerative diseases. Biomed. Pharmacother..

[10] Abdolmaleki S., Aliabadi A., Khaksar S. (2025). Bridging the gap between theory and treatment: Transition metal complexes as successful candidates in medicine. Coord. Chem. Rev..

[11] Duncan C., White A.R. (2012). Copper complexes as therapeutic agents. Metallomics.

[12] Avdović E., Dimić D., Milenković D. (2020). Investigation of Coumarin Derivative 3-(1-o-Toluidinoethylidene)-chromane-2,4-dione: IR Spectroscopic Characterization, NBO, and AIM Analysis and Molecular Docking Studies.

[13] Milenković D., Avdović E., Dimić D., Sudha S., Ramarajan D., Trifunović S., Marković Z.S. (2020). Vibrational and Hirshfeld surface analyses, quantum chemical calculations, and molecular docking studies of coumarin derivative 3-(1-m-toluidinoethylidene)-chromane-2,4-dione and its corresponding palladium(II) complex. J. Mol. Struct..

[14] Hu F., Zhang L., Nandakumar K.S., Cheng K. (2021). Imidazole scaffold-based compounds in the development of therapeutic drugs. Curr. Top. Med. Chem..

[15] Kumar N., Goel N. (2023). Recent development of imidazole derivatives as potential anticancer agents. Phys. Sci. Rev..

[16] Chopra B., Dhingra A.K., Prasad D.N. (2020). Imidazole: An emerging scaffold showing its therapeutic voyage to develop valuable molecular entities. Curr. Drug Res. Rev..

[17] Kasperkiewicz K., Ponczek M.B., Owczarek J., Guga P., Budzisz E. (2020). Antagonists of vitamin K—Popular coumarin drugs and new synthetic and natural coumarin derivatives. Molecules.

[18] Oxford Diffraction/Agilent Technologies UK Ltd. (2022). CrysAlisPRO.

[19] (2012). SAINT.

[20] Sheldrick G.M. *SADABS, Program for Empirical Absorption Correction of Area Detector Data*; University of Göttingen: Göttingen, Germany, 1996. https://api.semanticscholar.org/CorpusID:221929729.

[21] Sheldrick G.M. (2015). SHELXT–Integrated space-group and crystal-structure determination. Acta Crystallogr. A Found. Adv..

[22] (2012). European Committee on Antimicrobial Susceptibility Testing—EUCAST. http://www.eucast.org.

[23] Coppola G.M., Dodsworth R.W. (1981). Synthetic applications of α,β-unsaturated carbonyl compounds. Synthesis.

[24] Etter M.C. (1990). Encoding and Decoding Hydrogen-Bond Patterns of Organic Compounds. Acc. Chem. Res..

[25] Makowska A., Sączewski F., Bednarski P.J., Gdaniec M., Balewski Ł., Warmbier M., Kornicka A. (2022). Synthesis, Structure and Cytotoxic Properties of Copper(II) Complexes of 2-Iminocoumarins Bearing a 1,3,5-Triazine or Benzoxazole/Benzothiazole Moiety. Molecules.

[26] Mahfouz M.S., Ali A.A.M., Shebl M., Adly O.M.I. (2025). Fouad Copper(II) Chelates of a Coumarin-Based Acyl Hydrazone Ligand: Structural Characterization and Computational Evaluations for Prospective Applications in Antimicrobial, Antiviral, Antioxidant, and Anticancer Therapies. RSC Adv..

[27] Emami S., Dadashpour S. (2015). Current developments of coumarin-based anticancer agents in medicinal chemistry. Eur. J. Med. Chem..

[28] Jevtić M., Stanojević Pirković M., Komazec T., Mojić M., Mijatović S., Maksimović-Ivanić D., Dimić D., Marković Z., Simijonović D., Milenković D. (2024). A comprehensive evaluation of a coumarin derivative and its corresponding palladium complex as potential therapeutic agents in the treatment of gynecological cancers: Synthesis, characterization, and cytotoxicity. Pharmaceutics.

[29] Yadav A.K., Maharjan Shrestha R., Yadav P.N. (2024). Anticancer mechanism of coumarin-based derivatives. Eur. J. Med. Chem..

[30] Rogalewicz B., Czylkowska A. (2025). Recent Advances in the Discovery of Copper(II) Complex-es as Potential Anticancer Drugs. Eur. J. Med. Chem..

[31] Lu W., Tang J., Gu Z., Sun L., Wei H., Wang Y., Yang S., Chi X., Xu L. (2023). Crystal Structure, In Vitro Cytotoxicity, DNA Binding and DFT Calculations of New Copper(II) Complexes with a Coumarin–Amide Ligand. J. Inorg. Biochem..

[32] Ballazhi L., Imeri F., Jashari A., Popovski E., Stojković G., Dimovski A.J., Mikhova B., Mladenovska K. (2017). Hydrazinyldiene-chroman-2,4-diones in inducing growth arrest and apoptosis in breast cancer cells: Synergism with doxorubicin and correlation with physicochemical properties. Acta Pharm..

[33] Joshi C., Chejara P., Mahajan D.H. (2020). Facile synthesis and characterization of some new 5-ethylidene-thiazolidine-2,4-diones and their antimicrobial evaluation. Indian J. Pharm. Sci..

[34] Wu Y., Xu J., Liu Y., Zeng Y., Wu G. (2020). A review on anti-tumor mechanisms of coumarins. Front. Oncol..

[35] Dimić D.S., Marković Z.S., Saso L., Avdović E.H., Đorović J.R., Petrović I.P., Stanisavljević D.D., Stevanović M.J., Potočňák I., Samoľová E. (2019). Synthesis and characterization of 3-(1-((3,4-dihydroxyphenethyl)amino)ethylidene)-chroman-2,4-dione as a potential antitumor agent. Oxid. Med. Cell. Longev..

[36] Wu D.-L., Liao Z.-D., Chen F.-F., Zhang W., Ren Y.-S., Wang C.-C., Chen X.-X., Peng D.-Y., Kong L.-Y. (2019). Benzophenones from Anemarrhena asphodeloides Bge. exhibit anticancer activity in HepG2 cells via the NF-κB signaling pathway. Molecules.

[37] Kumar P., Asati V., Choubey A. (2021). Synthesis and characterization of novel 3-(aminomethyl)-5-benzylidenethiazolidine-2,4-dione derivatives as anticancer agents. J. Appl. Sci. Res..

[38] Nastasă C., Tamaian R., Oniga O., Tiperciuc B. (2019). 5-Arylidene(chromenyl-methylene)-thiazolidinediones: Potential new agents against mutant oncoproteins K-Ras, N-Ras and B-Raf in colorectal cancer and melanoma. Medicina.

[39] Chothani S.R., Chamakiya C.A., Joshi R.J., Karmur M.B., Karmur S.B., Varu H.L., Pissurlenkar R.R.S., Patel A.S., Kapuriya N.P. (2024). Synthesis, anticancer evaluation and in silico studies of novel N-substituted arylidenethiazolidine-2,4-dione derivatives as adenosine monophosphate-activated protein kinase activators. J. Heterocycl. Chem..

[40] Abraham M.H., Acree W.E. (2017). Descriptors for Pentane-2,4-dione and Its Derivatives. J. Solution Chem..

[41] Asati V., Bharti S.K., Rathore A., Mahapatra D.K. (2017). SWFB and GA Strategies for Variable Selection in QSAR Studies for the Validation of Thiazolidine- 2,4-Dione Derivatives as Promising Antitumor Candidates. Indian J. Pharm. Educ. Res..

[42] Mutebi J.K., Wu C.-C., Fang C.-Y., Hsu T.-K., Lin I.-C., Huang S.-W., Chiu Y.-C., Hsu B.-M. (2025). Exploring the impact of chemotherapy on the emergence of antibiotic resistance in the gut microbiota of colorectal cancer patients. Antibiotics.

[43] Safdar A., Bodey G., Armstrong D. (2011). Infections in patients with cancer: Overview. Principles and Practice of Cancer Infectious Diseases.

[44] Karcz D., Starzak K., Ciszkowicz E., Lecka-Szlachta K., Kamiński D., Creaven B., Jenkins H., Radomski P., Miłoś A., Ślusarczyk L. (2021). Novel coumarin–thiadiazole hybrids and their Cu(II) and Zn(II) complexes as potential antimicrobial agents and acetylcholinesterase inhibitors. Int. J. Mol. Sci..

[45] Yernale N.G., Mathada M.B.H. (2020). Preparation of octahedral Cu(II), Co(II), Ni(II), and Zn(II) complexes derived from 8-formyl-7-hydroxy-4-methylcoumarin: Synthesis, characterization and biological study. Mol. Struct..

[46] Sunitha N., Raj C.I.S., Kumari B.S. (2023). Synthesis, spectral studies, biological evaluation and molecular docking studies of metal complexes from coumarin derivative. Mol. Struct..

[47] Abdel-Kader N.S., Moustafa H., El-Ansary A.L., Sherif O.E., Farghaly A.M. (2021). A coumarin Schiff base and its Ag(I) and Cu(II) complexes: Synthesis, characterization, DFT calculations and biological applications. New J. Chem..

