# Supplementary material for: Synthesis, Crystal Structure, Antitumor, and Antimicrobial Activity of Novel Copper(II) Complexes with a Coumarin Derivative Containing a Histamine Substituent
Source: Molecules. 2026 Jan 1;31(1):162. doi: 10.3390/molecules31010162 (PMC12788114; doi:10.3390/molecules31010162)

Supplementary Materials

Synthesis, crystal structure, antitumor and antimicrobial activity of novel copper(II) complexes with a coumarin derivatives containing a histamine substituent

Ewelina Namiecińska [1], Pawel Hikisz [2], Patryk Czapnik [3,4], Magdalena Malecka [3], Magdalena Grazul [5], Peter Mayer [6], Ingo-Peter Lorenz [6], Elzbieta Budzisz [1,*]

Table S1. The antimicrobial activity (expressed as MIC and MBC) of metal complexes **3a**–**3c** and chosen antibiotics.

The species	Ligand 2a [µM] MIC/MBC	Ligand 2b [µM] MIC/MBC	Ligand 2c [µM] MIC/MBC	Complex 3a [µM] MIC/MBC	Complex 3b [µM] MIC/MBC	Complex 3c [µM] MIC/MBC	VAN [µM] MIC/MBC	TZP [µM] MIC/MBC
***E. coli* ATCC8739**	> 500	> 500	> 500	> 500	> 500	> 500	-	6.66
***E. coli* ATCC10539**	> 500	> 500	> 500	> 500	> 500	> 500	-	6.66
***E. coli* ATCC25922**	> 500	> 500	> 500	> 500	> 500	> 500	-	6.66
***E. faecalis* ATCC29212**	> 500	> 500	> 500	> 500	> 500	> 500	-	23.32
***E. faecalis* PCM896**	> 500	> 500	> 500	> 500	> 500	> 500	-	23.32
***E. faecalis* ATCC1299**	> 500	> 500	> 500	> 500	> 500	> 500	-	23.32
***E. faecium* PCM1859**	> 500	> 500	> 500	> 500	> 500	> 500	-	23.32
***P. aeruginosa* ATCC15442**	> 500	> 500	> 500	> 500	> 500	> 500	-	26.64
***P. vulgaris* CCM1799**	> 500	> 500	> 500	> 500	> 500	> 500	-	0.67
***S. epidermidis* ATCC12228**	>500	>500	>500	> 500	78.6/78.6	>500	0.35	-
***S. aureus* ATCC6538**	>500	>500	>500	> 500	78.6/78.6	>500	0.35	-

Figure 1S Spectrum FTIR (KBr cm^{-1}) for compound **2a:**

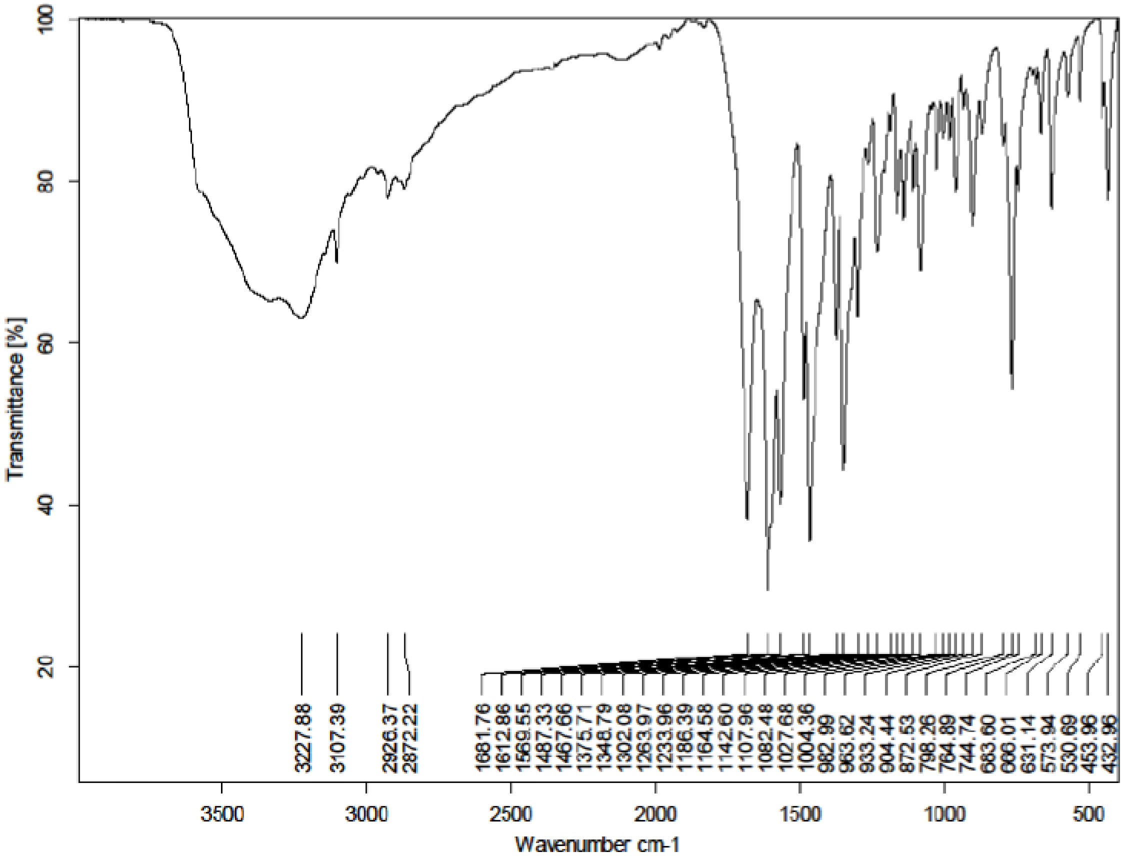

Figure 2S Spectrum FTIR (KBr cm^{-1}) for compound **2b:**

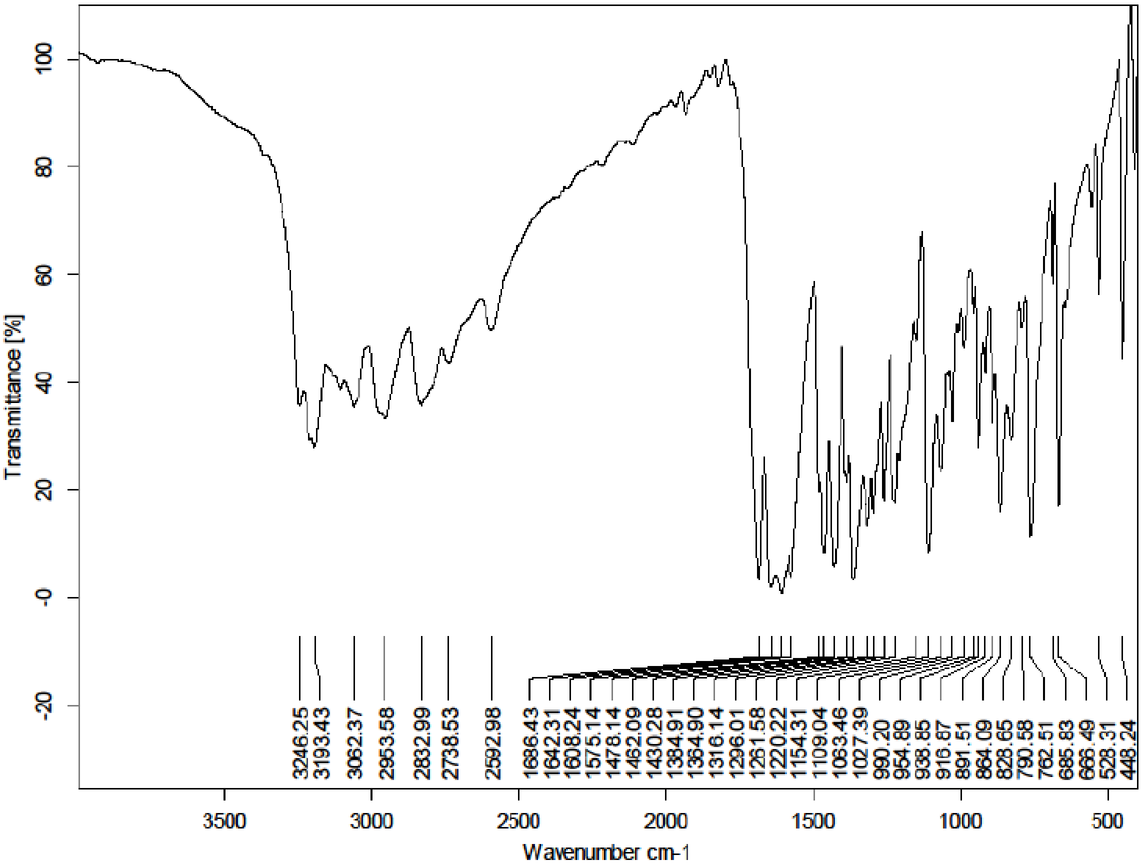

Figure 3S Spectrum FTIR (KBr cm^{-1}) for compound **2c:**

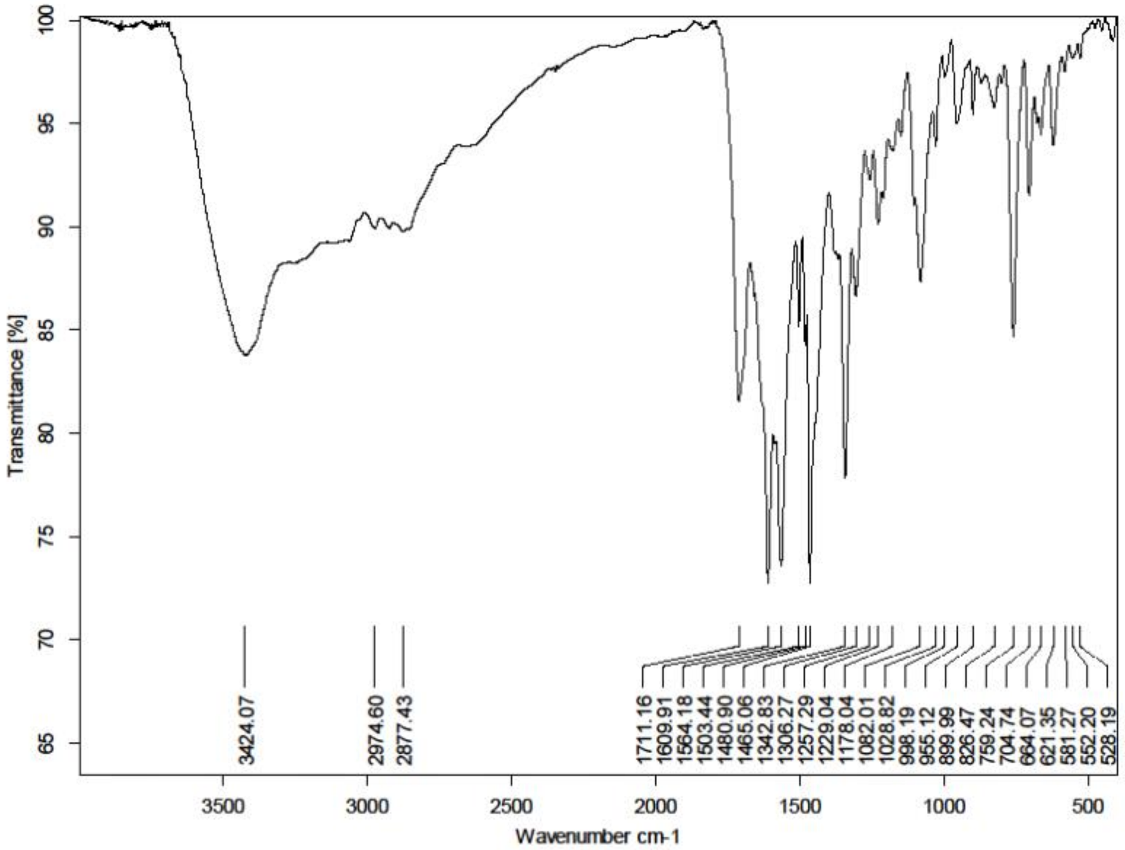

Figure 4S Spectrum ^{1}H NMR (600 MHz, DMSO-d^6) for compounds **2a:**

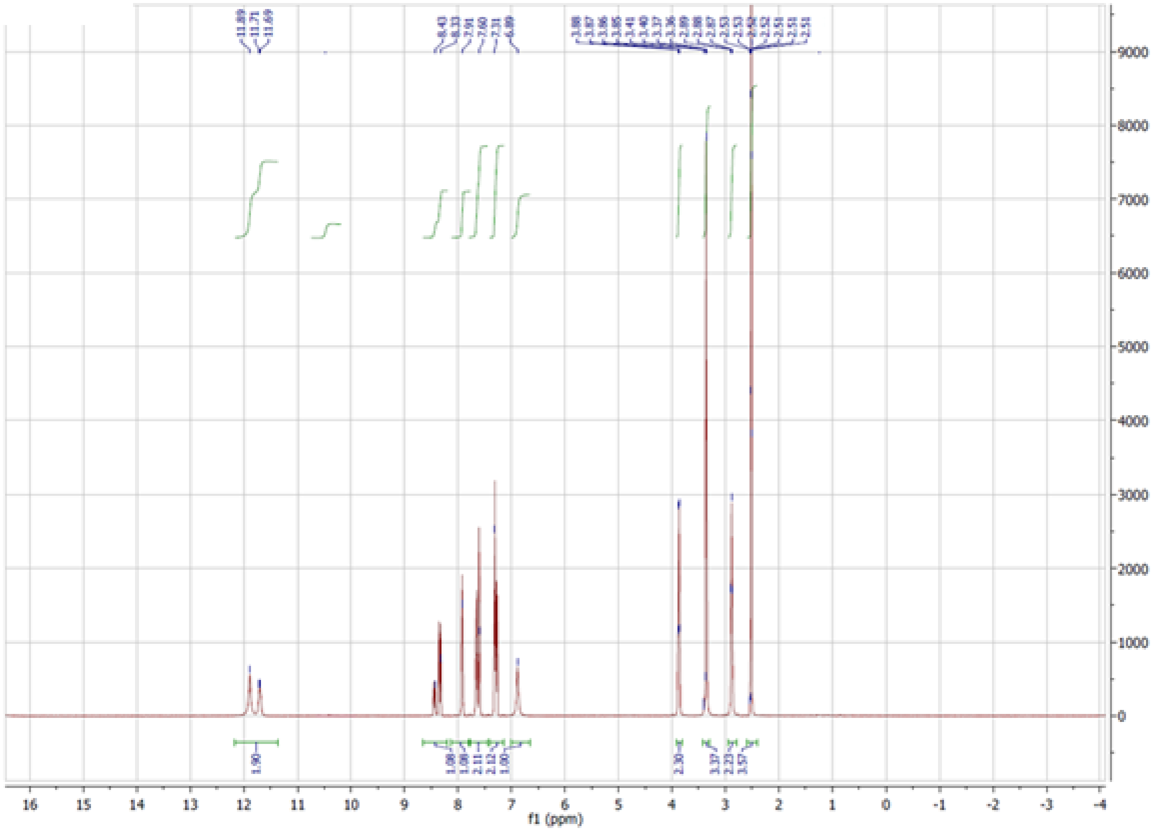

Figure 5S Spectrum ^{1}H NMR (600 MHz, DMSO-d[6]) for compound **2b:**

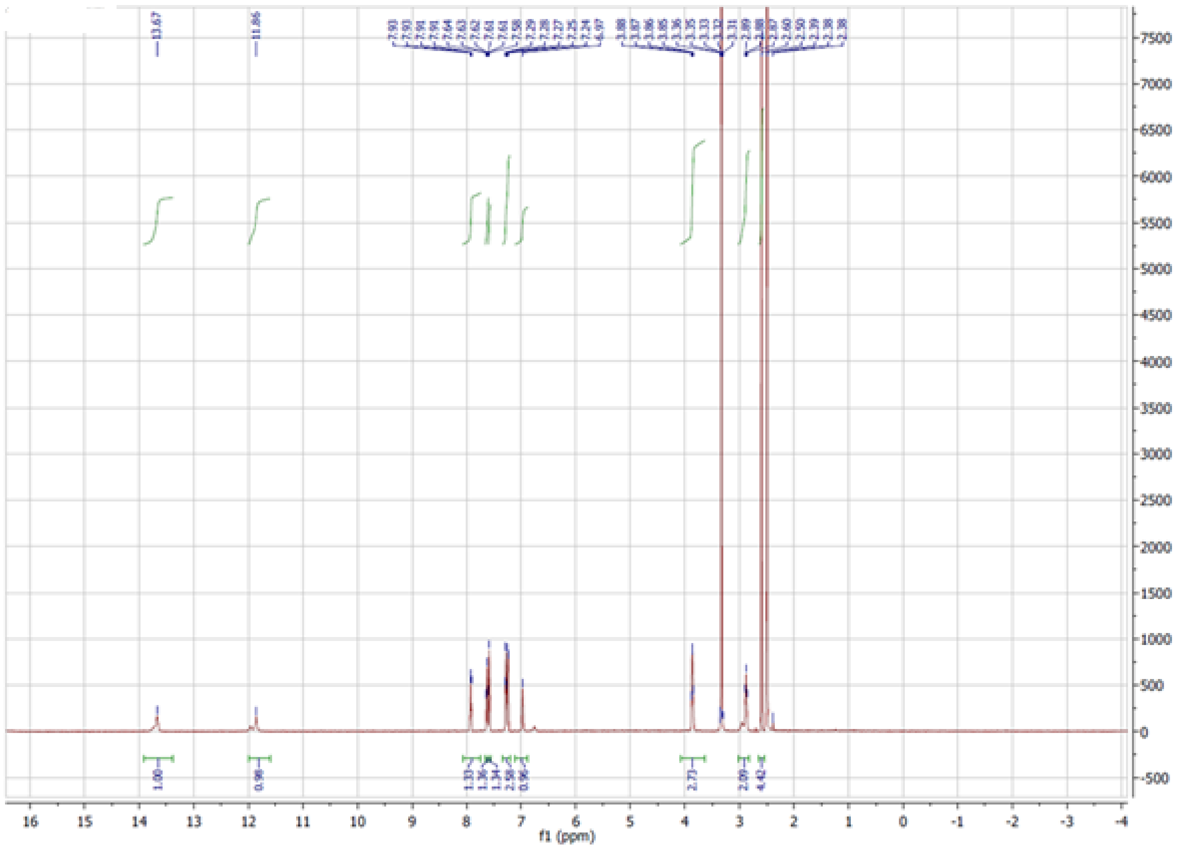

Figure 6S Spectrum ^{1}H NMR (600 MHz, DMSO-d^{6}) for compound **2c:**

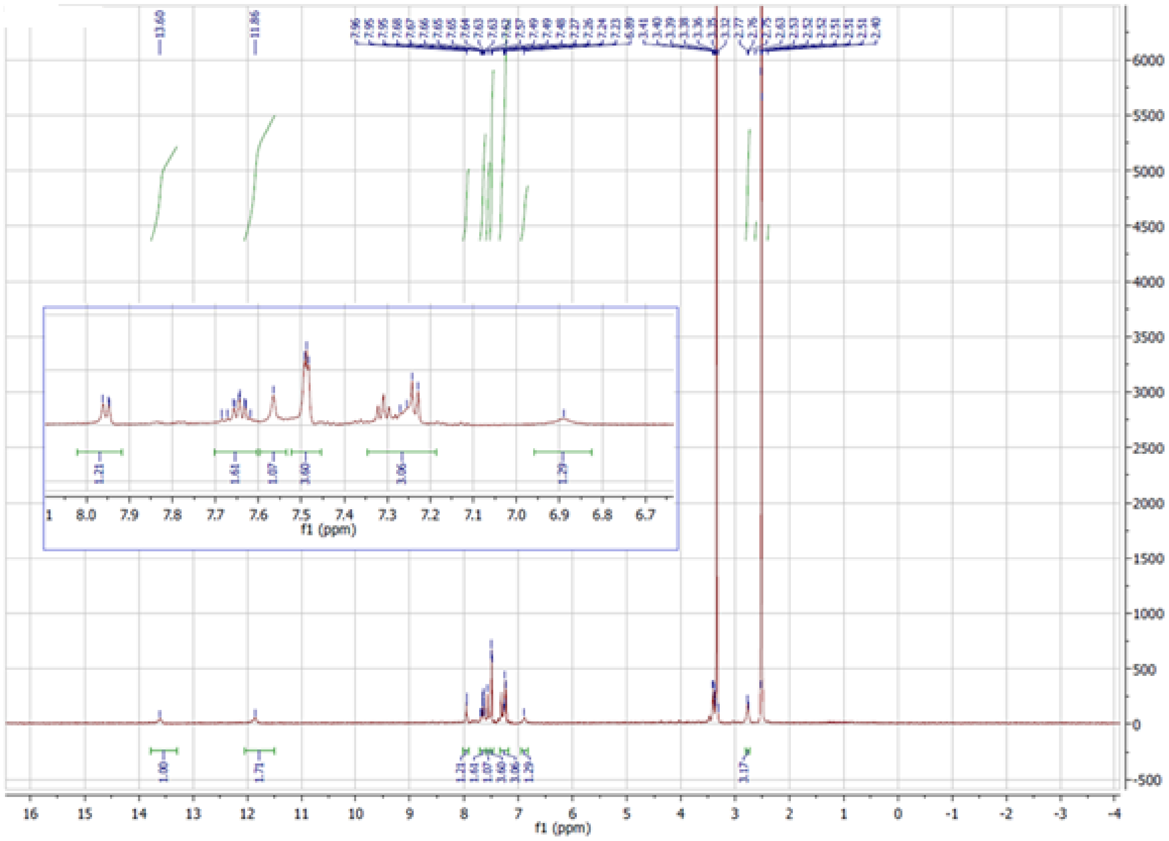

Figure 7S Spectrum FTIR (KBr cm^{-1}) for compound **3a:**

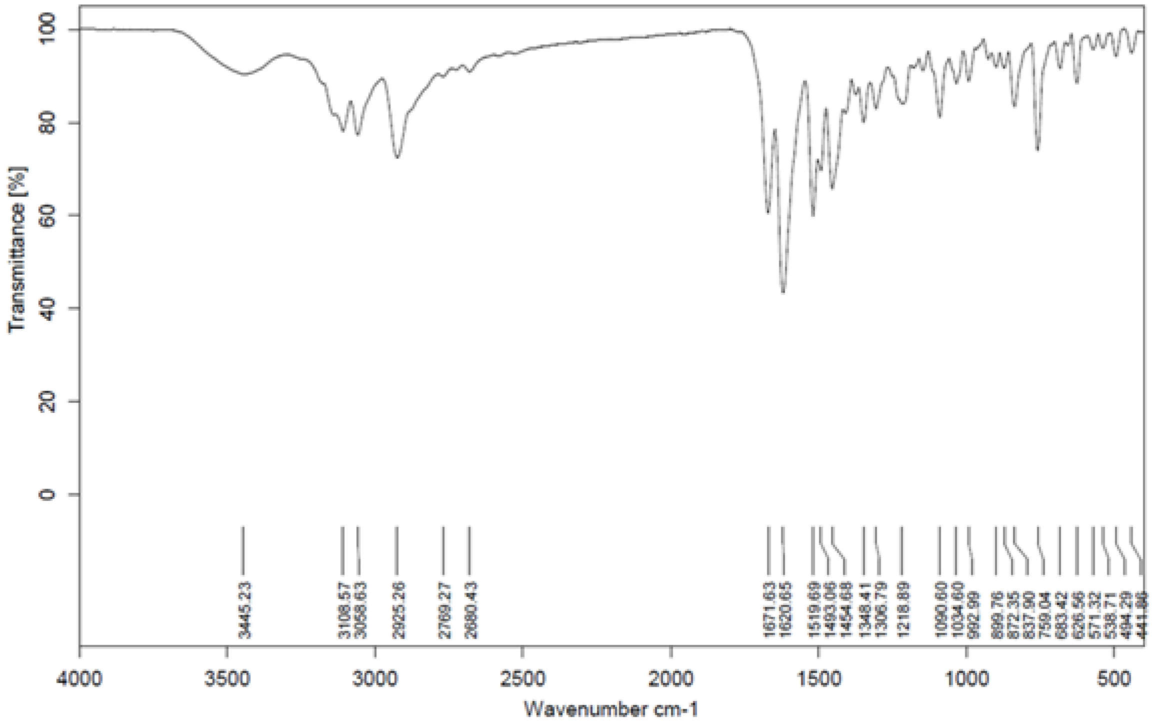

Figure 8S Spectrum FTIR (KBr cm^{-1}) for compound **3b:**

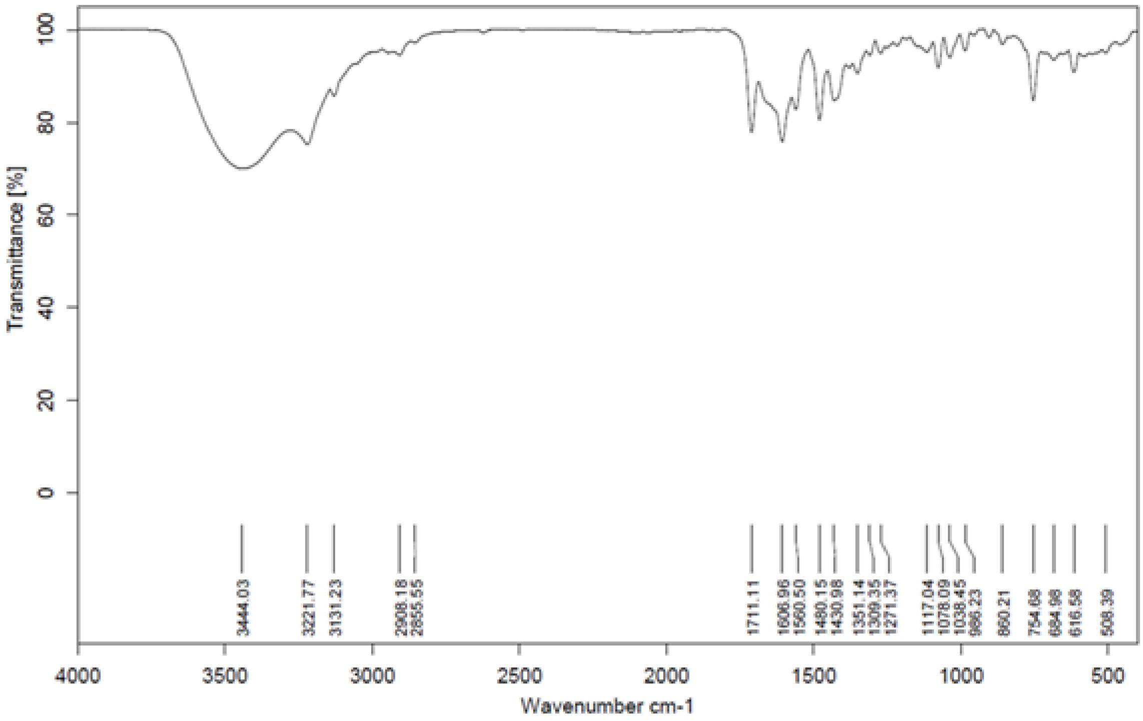

Figure 9S Spectrum FTIR (KBr cm^{-1}) for compound **3c:**

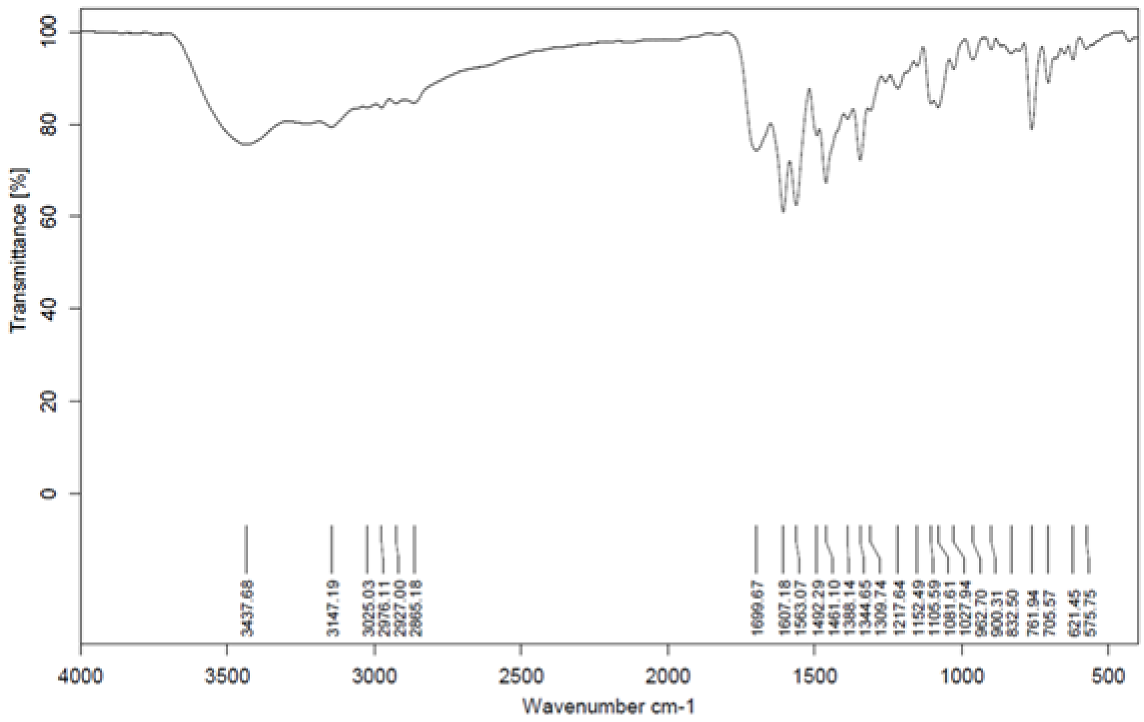

Figure 10aS ESI mass spectra of compound **3a** in positive ion mode:

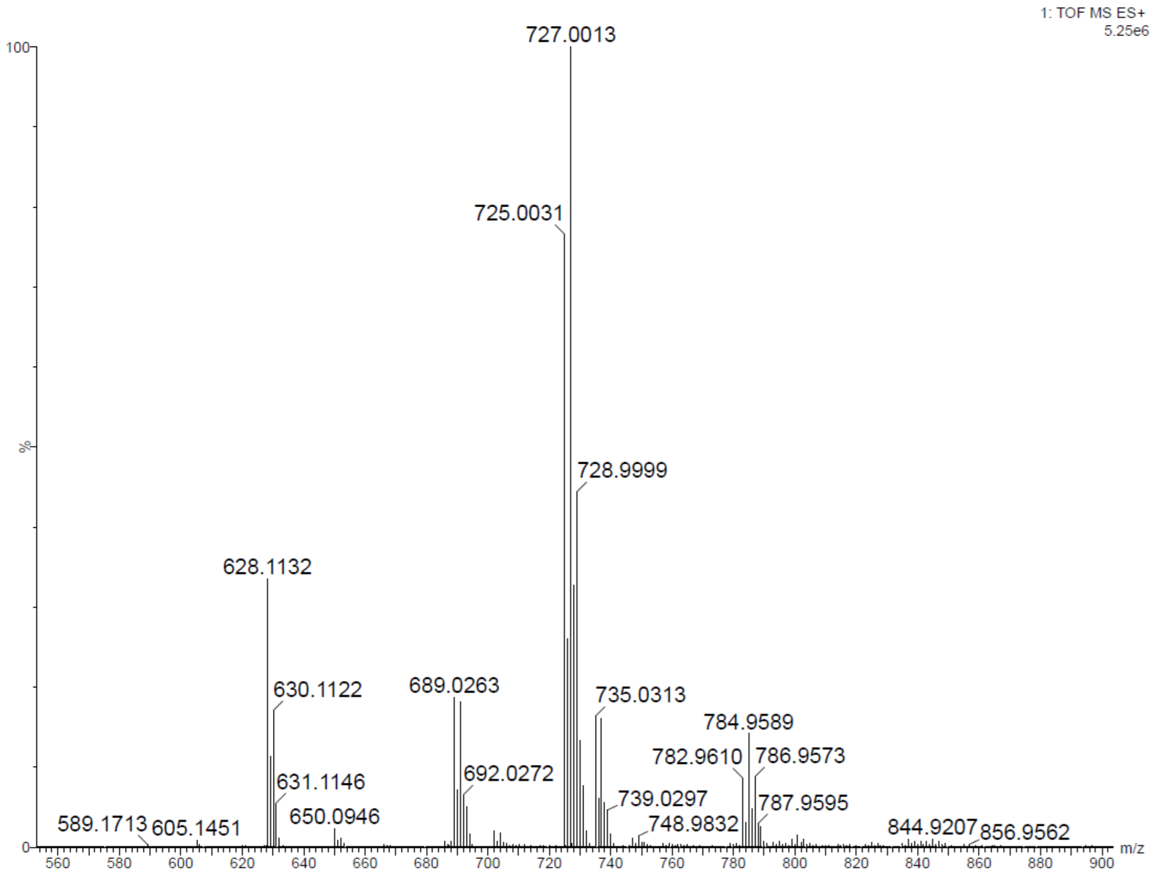

Figure 11aS ESI mass spectra of compound **3b** in negative ion mode:

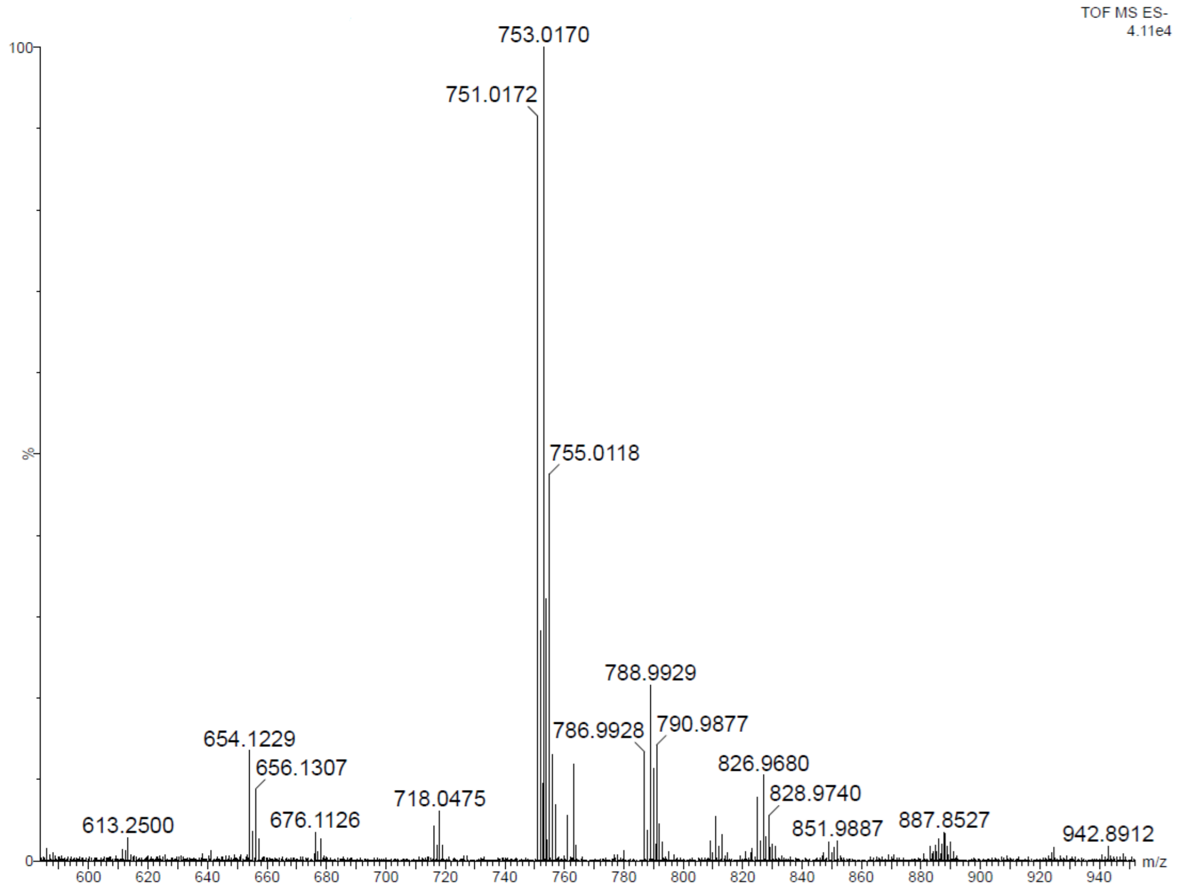

Figure 11bS ESI mass spectra of compound **3b** in positive ion mode:

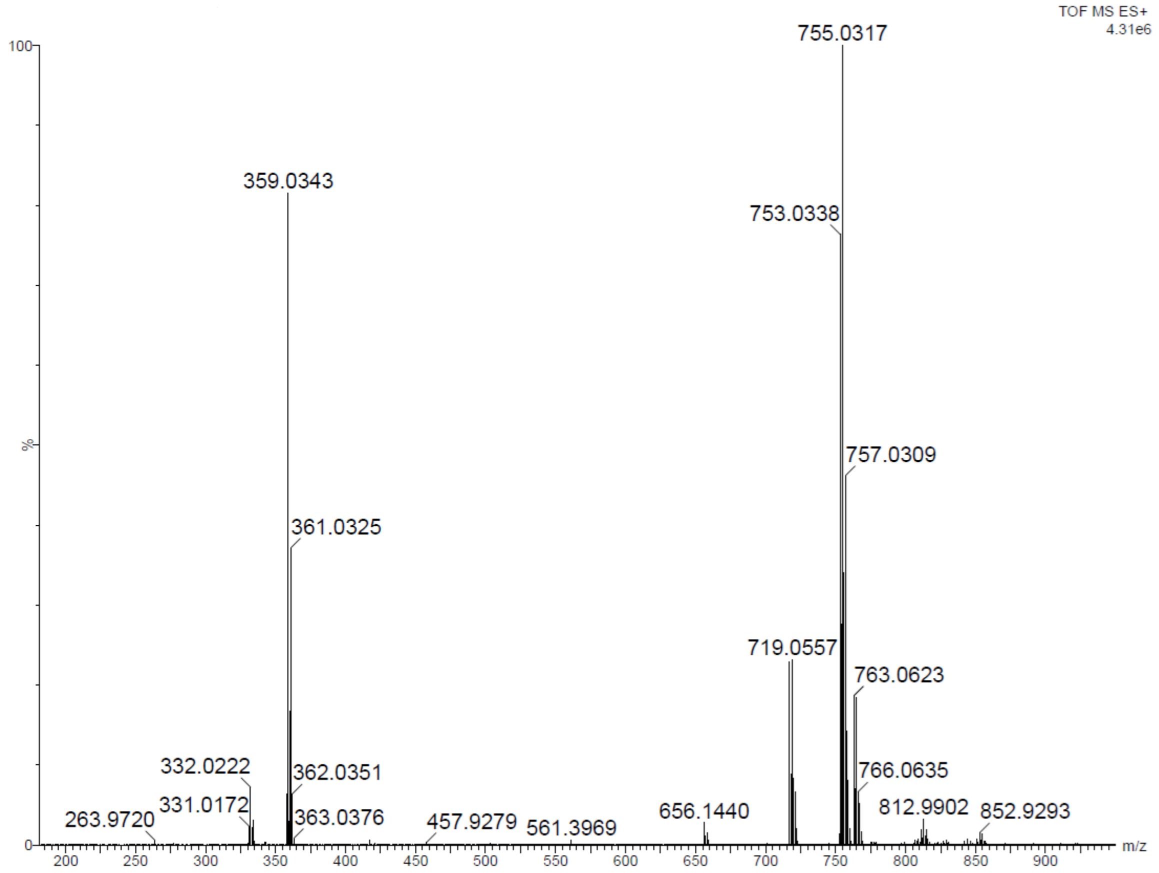

TOF MS ES+
4.31e6
755.0317
359.0343
753.0338
757.0309
361.0325
719.0557
763.0623
332.0222
362.0351
766.0635
263.9720
331.0172
363.0376
457.9279
561.3969
656.1440
812.9902
852.9293
100
%
0
200
250
300
350
400
450
500
550
600
650
700
750
800
850
900
m/z

Figure 11cS ESI mass spectra zoom of views of target ions for compound **3b** in positive ion mode:

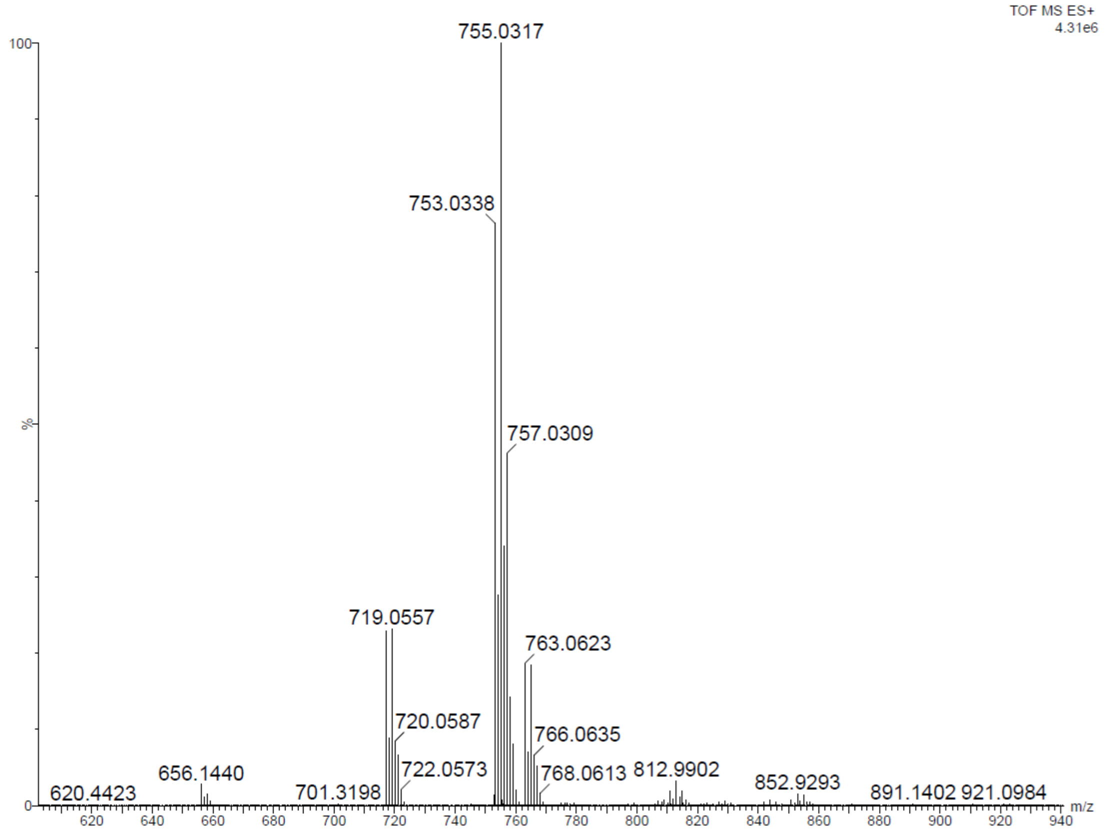

Figure 12aS ESI mass spectra of compound **3c** in negative ion mode with the experimentally observed peak at m/z 948:

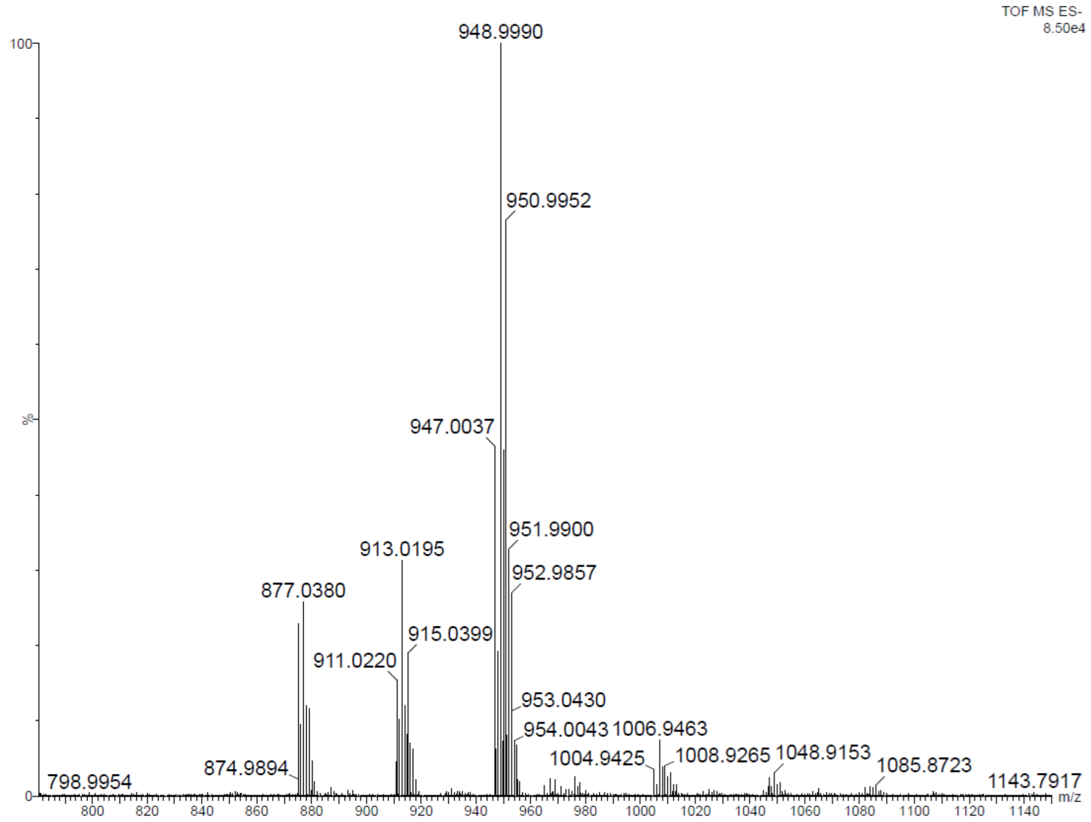

Figure 12bS ESI mass spectra of compound **3c** in negative ion mode with the experimentally observed peak at m/z 455:

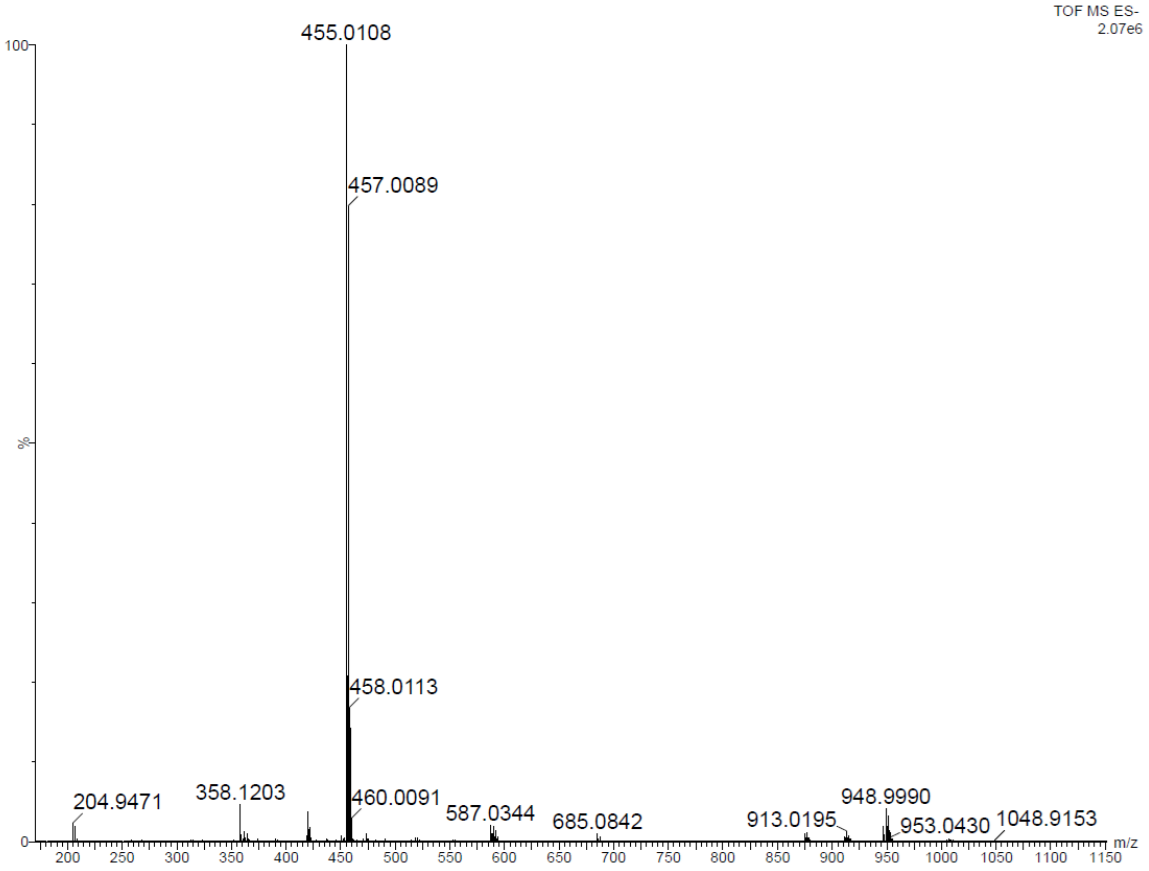

Figure 12cS ESI mass spectra of compound **3c** in negative ion mode with the experimentally observed peak at m/z 421:

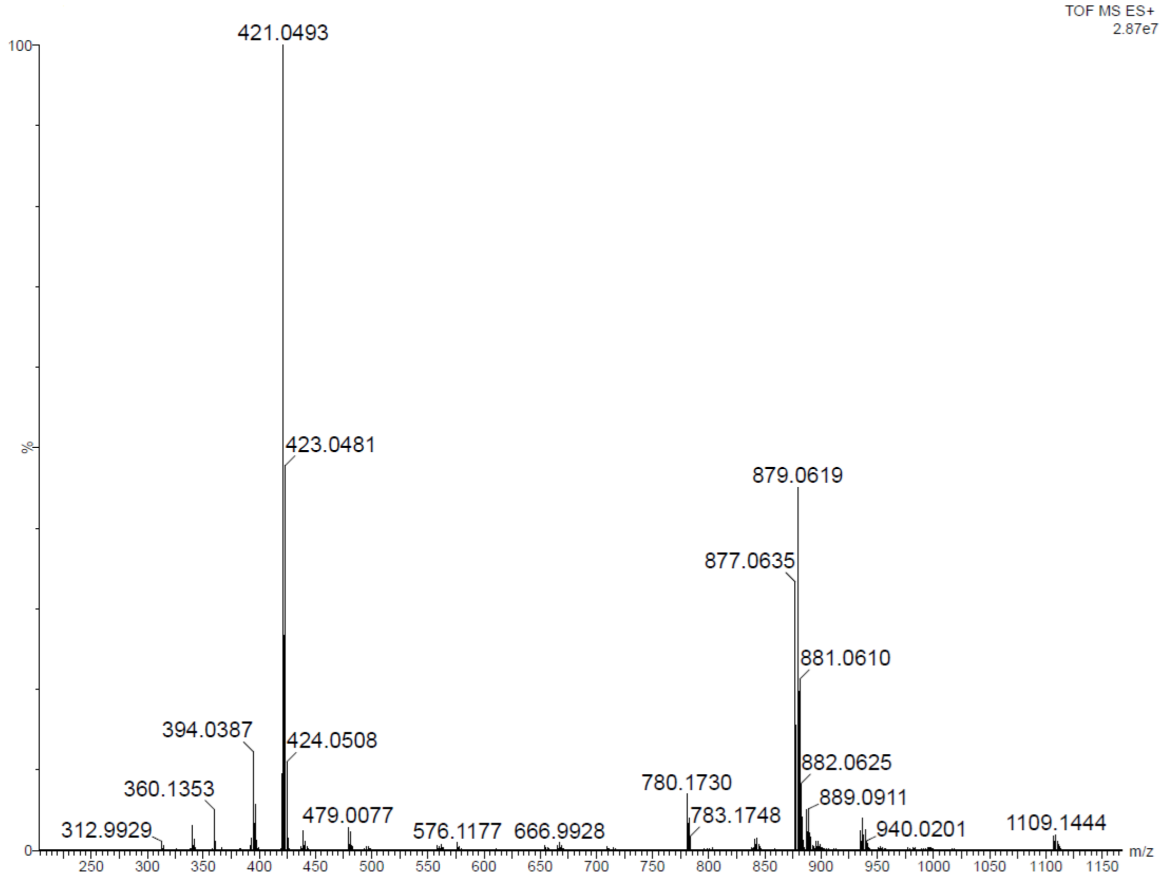

Figure 13S The simulated isotope patterns of the corresponding compound **3a:**

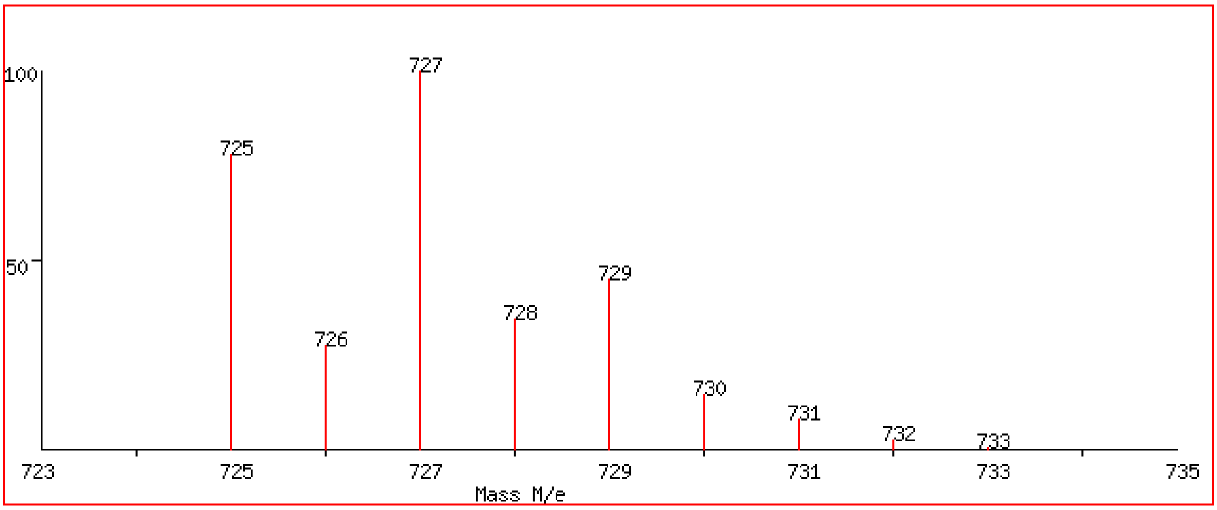

Figure 14S The simulated isotope patterns of the corresponding compound **3b:**

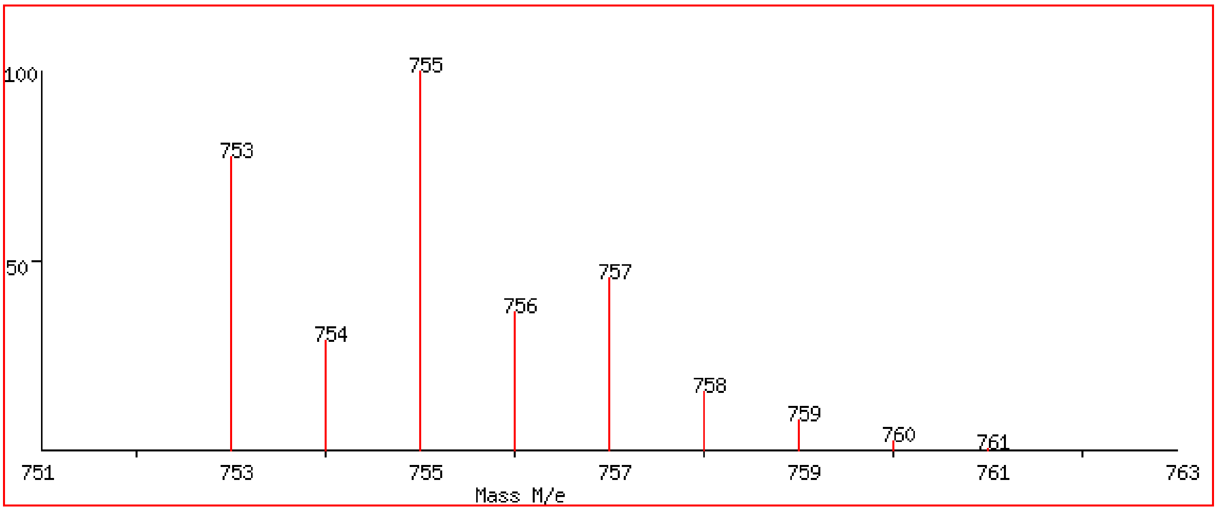

Figure 15aS The simulated isotope patterns of the corresponding compound **3c** after loss of crystallization water:

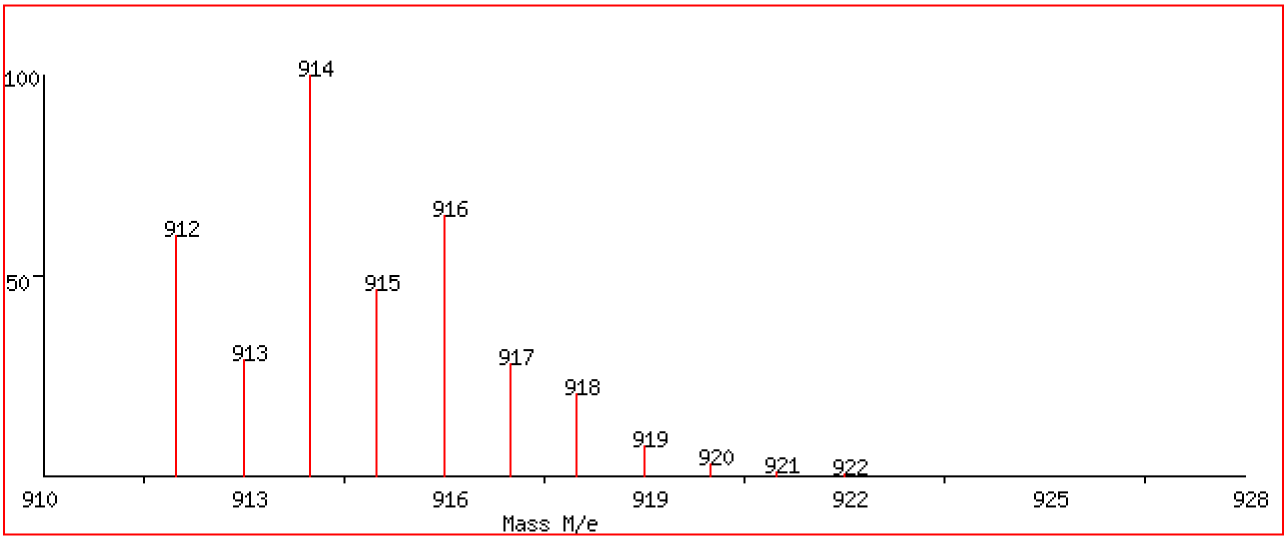

Figure 15bS The simulated isotope patterns of the corresponding ion of compound **3c**, after loss of crystallization water for the monomer under ESI:

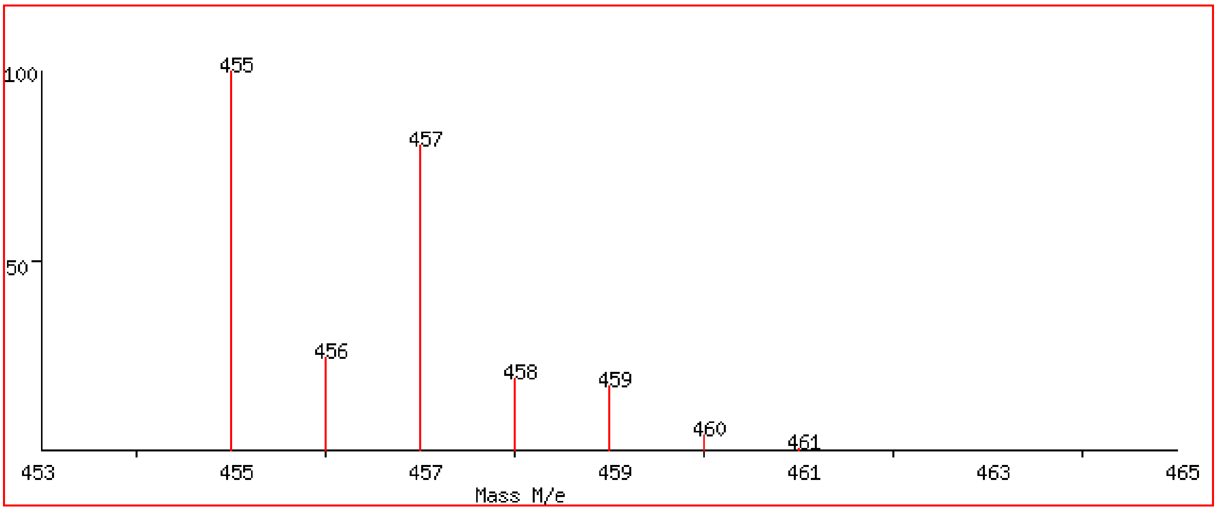

Supplement: Supplementary file 1 [file molecules-31-00162-s001.zip › molecules-4031819-supplementary.pdf]
